# Volume changes in white matter pathways from infancy to early adulthood measured using diffusion tensor based morphometry

**DOI:** 10.3389/fneur.2025.1624779

**Published:** 2025-09-12

**Authors:** Amritha Nayak, Rakibul Hafiz, M. Okan Irfanoglu, Carlo Pierpaoli

**Affiliations:** Laboratory on Quantitative Medical Imaging, National Institute of Biomedical Imaging and Bioengineering, Bethesda, MD, United States

**Keywords:** diffusion tensor imaging (DTI), brain development, morphometry, growth trajectory, pediatric, volume

## Abstract

**Background:**

Diffusion tensor imaging (DTI) has proven valuable in assessing structural and architectural features of white matter (WM) in postnatal development. Diffusion tensor-based morphometry (DTBM) uses DTI data to measure local volume changes and has been demonstrated in previous studies to be informative in the evaluation of specific WM pathways in several neurological disorders. In this study, we assess DTBM volume changes during postnatal brain development in typically developing children. In addition, we evaluate in each pathway the relationship between changes in volume and DTI metrics.

**Method:**

We included DTI data from 182 healthy participants in the age range of 0–21 years, from the publicly available database: the NIH Pediatric MRI Data (NIHPD). Data were processed using the TORTOISE pipeline and age-specific templates were created using the diffusion tensor-based registration tool DRTAMAS. Region of interests (ROIs) were defined on a study-specific, young-adult reference template (18–21 years). Individual brains were registered to the reference template using a two-step process involving age-specific templates. ROI values for volume and DTI metrics were normalized to the median values of the 18-21-year group. Developmental trajectories were analyzed in two age segments; Segment 1: data between 0 and 2.69 years and Segment 2: for the remaining age range.

**Results:**

The results show that volumetric developmental trajectories varied largely among WM regions. The estimated volume at birth ranged: 12–53% of the adult value; where the rate of growth ranged: 3–30% of the adult value per year, in Segment 1; and 0–4% afterwards (Segment 2). The Corticospinal Tract, for example, showed protracted growth into young adulthood, while the Corpus Callosum growth was almost completed in the first 3 years. The magnitude of changes in local volume were generally larger than the magnitude of changes in diffusion metrics. Moreover, volumetric changes were more protracted, i.e., for many regions volume continued to increase even when diffusion metrics had reached a plateau.

**Conclusion:**

In conclusion, DTBM has shown developmental trajectories for WM volume in the human brain that are pathway specific and distinct from those obtained for DTI metrics. In future studies, DTBM should be performed in larger cohorts to assess correlation with cognitive and behavioral changes as well as establish ranges for normative values.

## 1 Introduction

In postnatal development, there are large changes in brain composition, structure, morphology, and function. In clinical practice, the simple measurement of head circumference is used as a proxy to monitor brain development with respect to normative age and sex adjusted data. For clinical and research purposes, non-invasive mapping of brain features by MRI could provide additional more sensitive, specific, and biologically informative tools for evaluating brain development. Particularly, it would be helpful for clinicians to have reliable normative measurements of morphological features, such as local volume in different brain structures, to be used as a reference for assessing development in health and disease. These regional volume measures, cross-sectionally or longitudinally, in association with clinical functional scores could provide additional information to clinicians about effects of disease burden and disease progression. Magnetic resonance imaging can provide information about morphological features non-invasively (1–4), however, assessment in specific white matter pathways is complicated by two main factors: (1) In conventional T1 and T2 weighted images it is impossible to depict the boundaries of different white matter pathways, and (2) In the early stages of postnatal development segmenting white matter from gray matter is problematic on T1 and T2 weighted images because of the massive changes in contrast produced by myelination and hydration of the tissue (5).

Diffusion tensor imaging (DTI) is considered informative of structural/architectural features of biological tissues (6, 7). One advantage of DTI is that unlike on T1- and T2-weighted images (T1WI and T2WI, respectively), most WM tracts are easily identifiable even in an infant brain (8, 9). Studies have applied DTI and its metrics such as Fractional Anisotropy (FA), Mean Diffusivity (MD), Radial Diffusivity (RD), and Axial Diffusivity (AD) to investigate brain development from infancy to early childhood or adulthood (8, 10–24). Over the last decade, studies have utilized DTI tractography to segment white matter tracts and provide tract-specific measurements of DTI metrics along the length of the tract (12, 13, 16, 22, 25–29). However, DTI metrics *per se* are not informative of morphology. More recently, a few studies have performed tract-specific volumetric analysis in infants and young children using DTI tractography (12, 13, 22, 25, 28, 29). However, the extraction of the boundaries of specific pathways by tractography is greatly dependent on data quality, resolution, and other tracking criteria that could be challenging even in the fully developed adult brain (30, 31).

Diffusion-Tensor Based Morphometry (DTBM) (32) is a technique that extracts voxelwise volume differences by coregistering a source and a target DTI dataset. It has been shown that DTBM has higher sensitivity and regional specificity in capturing volume changes in white matter, compared to classical T1WI morphometry (T1-TBM) in various disease conditions (32–35). We performed DTBM in this study using DTI brain scans from infancy to early adulthood. The goals of this study were: (1) to provide a survey of the volumetric trajectory of different white matter pathways as measured by DTBM in typically developing children, (2) to compare our volumetric results with the results of DTI metrics in the same regions. To our knowledge, this is the first study that uses DTBM in evaluating morphological changes of brain structures in normal brain development.

## 2 Materials and methods

To accomplish the goals of the study, we adopted an experimental design with the following requirements: (a) selection of a study sample from infancy to adulthood, with an adequate number of subjects per age group, (b) data acquisition design, processing and quality control strategy that produces good quality DTI metrics and (c) a DTI registration and template creation pipeline that can handle large morphological changes across the age groups, to register brains of varying sizes to generate spatially coregistered brain structures for analysis.

### 2.1 Subjects

Our study population consisted of data from the expanded DTI (eDTI) protocol of the NIH Pediatric MRI Data database (NIHPD) (36). The study included 182 brain scans from 152 unique individuals ranging in ages from 0 to 21 years. Please refer to Supplementary Figure S.1 for details regarding the age group distribution and number of subjects within each group.

### 2.2 Image acquisition and processing

The image acquisition for the eDTI protocol was designed with adequate b-values and directions with a goal of assessing brain scans from infants and young children with greater sensitivity. The image acquisition and processing are described elsewhere (36). Briefly, the eDTI protocol included b = 0 s/mm^2^; 9 images (GE), 10 images (Siemens), b = 100 s/mm^2^; 10 images, b = 300 s/mm^2^; 10 images, b = 500 s/mm^2^; 10 images, b = 800 s/mm^2^; 30 images, and b = 1,100 s/mm^2^; 50 images, with full brain coverage using 60 slices, 2.5 mm slice thickness, FOV = 240 mm with a 96 × 96 matrix resulting in 2.5 × 2.5 × 2.5 mm^3^ voxels. All data were processed using TORTOISE software (37) and the processed data went through rigorous quality control (36, 38). The DWI processing steps included: (1) correction of eddy and motion distortion artifacts, (2) correction of susceptibility induced EPI distortions, and (3) reorientation of the corrected data to its respective AC-PC structural reference image.

### 2.3 Reference template creation

To achieve accurate registration in all brain regions, specifically for the alignment of individual WM pathways, we employed DRTAMAS (36), a diffusion tensor-based diffeomorphic registration and atlas creation pipeline. A young adult template was initially generated using DRTAMAS using 38 subjects [male (*n* = 20, mean = 19.7, SD = 1.3 yrs.), and female (*n* = 18, mean = 19.8 yrs., SD = 1.2 yrs.)] within the 18–21-year age group, with an average age of 19.5 years. Subsequently, the diffusion tensors from all study subjects were diffeomorphically aligned to this reference template to enable voxelwise and ROI-wise analysis. The term young adult template and reference template will be used interchangeably for the rest of the paper.

### 2.4 Age specific group template creation

As described in the previous section, the goal was to evaluate the changes in volume and DTI metrics of individual brains across the different age groups relative to the values from the young adult template. Due to the large age range of the subjects included in the study, brain size differences were significant. To avoid potential misregistration due to such large morphometric differences, we adopted a two-step registration strategy: the first step involved the creation of age specific group templates and the second step involved the registration of these age-specific templates to the young adult template. We hypothesized that this approach would be less susceptible to registration local minima compared to the direct approach where very young brains are registered to the adult template. To achieve this two-step strategy, individual scans were first grouped to create nineteen age-specific groups. An individual template was created for each age-specific group using the registration strategies described in the previous section (39). Age-specific group templates were created initially in increments of 6 months until 2 years to capture the rapid changes in morphology and DTI metrics at the youngest ages, and thereafter age-specific group templates were created in increments of 1 year. Please refer to Supplementary Figure S.1 for details describing the age distribution and number of subjects included in each age group.

### 2.5 Creation of normalized volume, FA, MD, RD, and AD maps

The image registration process generates a transformation that maps the moving (source) image to the fixed (target) image. Therefore, in our two-step registration framework, two sets of transformations were computed: the first set mapping each individual brain onto its corresponding age-specific template and the second set mapping the age-specific template to the young adult template. Supplementary Figure S.2 provides a schematic representation of the template creation pipeline.

**Figure 1:**
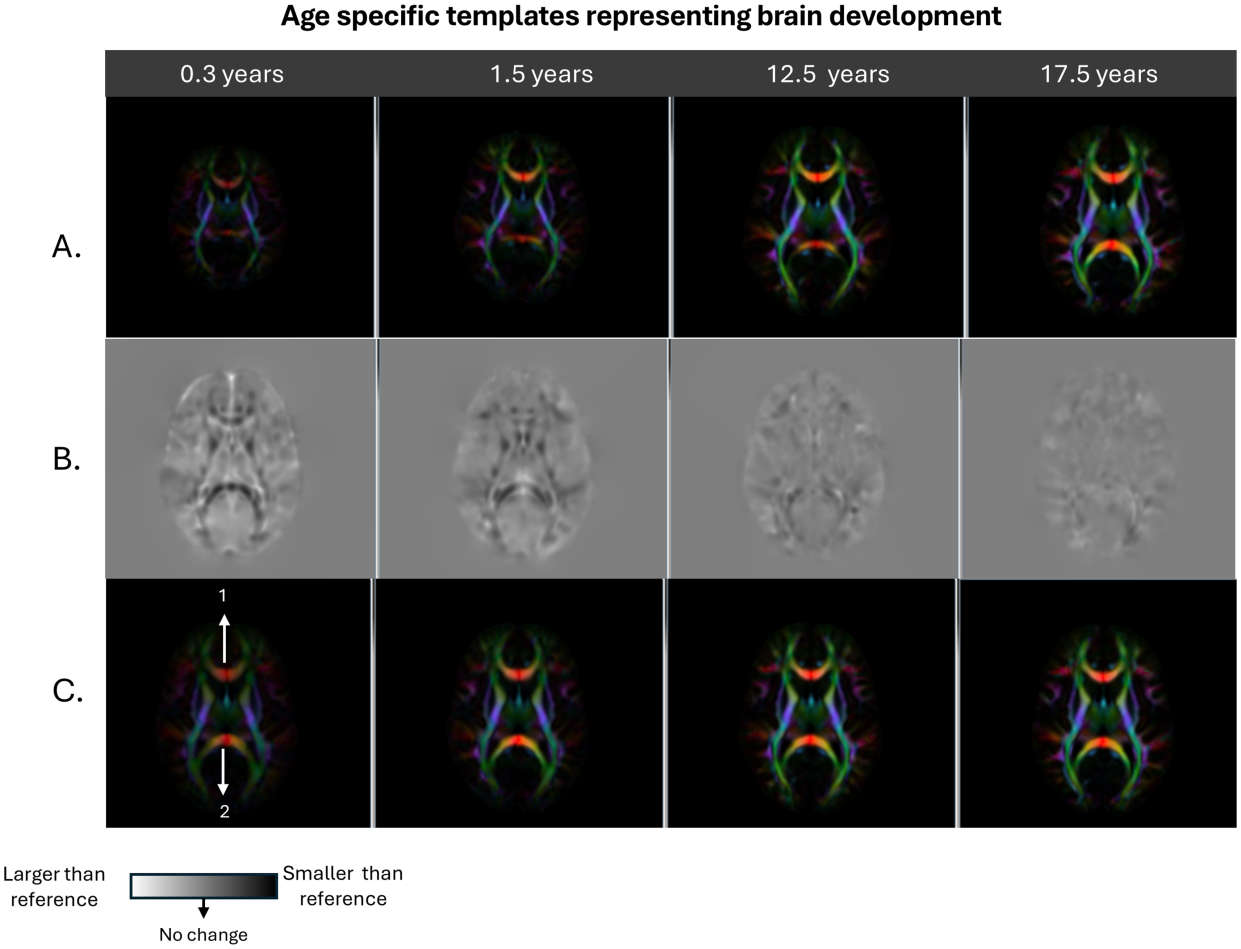
Displays examples of age specific average group templates: infancy (0.3 years), early childhood (1.5 years), beginning of adolescence (12.5 years), and end of adolescence (17.5 years). Row **A** shows the directionally encoded color (DEC) maps for each template prior to registration to the young adult reference template. Row **B** shows the determinant of the Jacobian maps generated from registering group templates to the reference templates which display the local size change information. Row **C** shows the final appearance of the group templates in the young adult template space. As can be observed from the Figure, Row **A** shows that there is a large difference in overall brain size between 0.3-year template and 1.5 year. The 12.5- and 17.5-year-old templates appear similar to each other, but are larger than the 0.3 – and 1.5-year-old templates. For the jacobian maps in Row **B**, dark voxels show regions that are smaller than the reference image and the bright voxels represent the opposite. The infant brain template has an overall brighter appearance compared with the gray background. The level of overall brightness is reduced considerably but still present in the Jacobian map generated for the 1.5-year-old template. Also, the Genu and Splenium of the Corpus Callosum, (structure locations shown with arrow number 1 and 2, respectively, in Row **C**, column 1) was apparent in the Jacobian map of the 0.3 year brain is less dark in the 1.5 year brain. It should be noted that the Jacobian maps appear a lot more homogenous with less structure in the 12.5- and 17.5-year-old brains.

These two transformations are subsequently combined to capture the overall mapping of individual brains to the adult template. The DTI-derived scalar maps, such as FA, MD, RD and AD were computed in the native space of the subjects, then warped onto the reference template space using the combined transformations, thereby normalizing all the DTI metrics across the study. Since the transformation map contains the amount of size and shape change a brain had to undergo to match the reference template, the volume of each brain and its local structures in relation to the adult brain could be assessed. In order to extract the quantitative volume change information, the Jacobian matrices of the combined transformation fields were first computed and the determinant of these voxelwise matrices were extracted to be used as a measure of local volume change. Figure 1 provides an example of a few salient age groups, showing large changes in brain morphometry and the resulting determinant of Jacobian maps. It should be noted that color brightness in the directionally encoded color maps is modulated by FA. Therefore, in very young brains the color brightness is low, with the brightness increasing with each age group, due to increasing FA with age.

It should also be noted that the Jacobian maps (row B of Figure 1) capture the morphometric changes various brain structures had to undergo to match the adult template. These features appear less detailed as age increases.

#### 2.5.1 Calculation of the whole brain volume maps

To measure the relative volume of the whole brain, the affine scaling component of the combined transformation is extracted and expressed as a relative percentage change of the adult brain as follows:


(1) $$\;\text{Global Volume}\;{\left(\Delta \%\right)}_{i}=\frac{e^{x_{i}}}{e^{\text{Median Reference}\;\mathrm{LnJ}\;\text{Value}}}\times 100$$

Where, i=1,2,3,…N Subjects, with $x_{i}$ representing the value of LnJ (logarithm of the determinant of the Jacobian) computed from the affine scaling component for the $i^{\mathrm{th}}$ subject, and the Median ReferenceLnJValue representing the median of the average LnJ values computed from the affine scaling component in the young adult subjects (reference template). *Note that while Jacobian (J) represents a change in volume, it cannot directly be used to perform mathematical or statistical operations because of its assymetric range which constrains it to be strictly positive due to diffeomorphism. Prior to any operations, it needs to be converted to LnJ, which is symmetrically distributed around zero.* Therefore, mathematical operations were performed after converting J to LnJ as described in Equation 1. Thereafter, the exponent with base e of the LnJ values was taken to obtain J again before computing the ratio to represent relative ‘volume’.

### 2.6 Regions of interest

With the registration steps detailed in section 2.4–2.5 we had achieved a voxel-wise correspondence between each subject and the reference template. Therefore, ROIs defined on the reference template can be used to extract measurements from the spatially normalized volume and DTI metrics maps. While the JHU (Johns Hopkins University) WM ROIs (40) are informative and readily available to be used in this type of analysis, it is defined on a single subject brain. Moreover, the ROIs are available only using registration to an FA template, with the ROIs larger in size and with some left and right discrepancies (41). The large ROIs can be problematic when extracting information from brains of very young subjects, because they may be more prone to partial volume effects, confounding the measurements for these very young age groups. Therefore, for this study, we used subcortical WM ROIs defined on a Human Connectome DTI template (42). These WM ROIs were drawn on the HCP template with the JHU WM ROIs as a reference but making sure that ROIs were restricted more to the core of the tract. Tensor based registration was performed between the HCP DTI template and the reference template for the study to transform the ROIs defined on the HCP template into the young adult template space using nearest neighbor interpolation. Putamen and Thalamus ROIs were defined on the young adult template. Due to anatomical variability, diffeomorphism in image registration is violated and accurate registration of data in the cortical folding cannot be achieved. Therefore, the only gray matter regions that could be reasonably assessed are the deep gray matter regions included in this study, namely, Putamen and Thalamus. These regions are added as control regions and were appropriate to be included due to reliability of registration in these regions compared to the cortex. Please refer to Supplementary Figure S.3 for information regarding the anatomical locations of the ROIs used in the analysis.

Thirty-four WM ROIs (inclusive of left and right structures) used for the study are grouped under commissural, associative, projection, and cerebellar pathways. The following section details the structures categorized under each pathway.

*Commissural*: Genu, Body, and Splenium of the Corpus Callosum, and Pontine Crossing Tract.

*Association*: External Capsule, Cingulum Cingulate Gyrus, Cingulum Hippocampus, Fornix Cres Stria Terminalis, Fornix, Superior Longitudinal Fasciculus, Superior Fronto-Occipital Fasciculus, Uncinate Fasciculus.

*Projection*: Cerebral Peduncle, Posterior Limb of Internal Capsule, Retro-lenticular part of Internal Capsule, Corticospinal Tract, and Medial Lemniscus.

*Cerebellar*: Inferior, superior, and middle cerebellar peduncle.

### 2.7 Calculation of ROI-wise measurements from normalized volume and DTI metric maps

#### 2.7.1 Relative change in FA, MD, RD, and AD

The transfer of ROIs into the young adult template and the spatial normalization of the FA, MD, RD, and AD maps into the template space allowed for the extraction of mean ROI values (43) for each metric. Thereafter, the values were expressed as a relative percentage with respect to the reference template to generate quantitatively normalized values as follows:


(2) $$\;\mathrm{DTI}\;{\left(\Delta \%\right)}_{i,j}=\frac{x_{i,j}}{{\text{Median Reference Value}}_{j}}\times 100$$

Where, i=1,2,3,…N Subjects and j=1,2,3,…M ROIs, with $x_{i,j}$ representing the average metric value within the $j^{\mathrm{th}}$ ROI for the $i^{\mathrm{th}}$ subject, and the ${\text{Median Reference Value}}_{j}$ representing the median of the average metric values computed for the $j^{\mathrm{th}}$ ROI, among young adults (reference template).

#### 2.7.2 Calculation of local structure volume

The Jacobian maps derived from the combined transformation, described in section 2.5 of this paper, also contain information about local volume changes for the structures to match the adult template. The same strategy used to calculate the mean ROI values can be used to extract volume information for structures from these maps. Due to reasons mentioned in section 2.5.1, and similar to Equation 1, to calculate the mean ROI values, the determinant of the Jacobian was converted to log of the determinant of the Jacobian (LnJ) as described in Equation 3, below. Thereafter, the average ROI values were computed using the same strategy as for the DTI metrics.

Similar to Equation 2, the average ROI-wise LnJ values were normalized with respect to the adult brain as follows:


(3) $$\;\text{Volume}\;{\left(\Delta \%\right)}_{i,j}=\frac{e^{x_{i,j}}}{e^{\text{Median Reference}\;\mathrm{LnJ}\;{\text{Value}}_{j}}}\times 100$$

Where, i=1,2,3,…N Subjects and j=1,2,3,…M ROIs, with $x_{i,j}$ representing the average value of LnJ within the $j^{\mathrm{th}}$ ROI for the $i^{\mathrm{th}}$ subject, and the $\text{Median Reference}\;\mathrm{LnJ}\;{\text{Value}}_{j}$ representing the median of the average LnJ values computed for the $j^{\mathrm{th}}$ ROI, among young adults (reference template). Note, the exponent with base e of these values was taken before computing the ratio to represent relative ‘volume’.

### 2.8 Statistical analysis

From visual observation, the percentage change in whole brain volume per subject, with respect to age, shows a nonlinear growth trajectory with a steep increase in volume in the initial years from birth, followed by a flattening of the trajectory (refer to Supplementary Figure S.4a). The ‘change-point’ in the trajectory of whole brain volume growth can be identified using two linear regressions (refer to section S.4 in the supplement). This approach allowed for a data driven approach of dividing the data into groups informed by the behavior of the growth trajectory of the whole brain volume across the age span of the study. Following this, ROI values, which were quantitively normalized from DTI metrics and for volume, were split into two groups; segment 1 before the ‘change-point’ (< 2.69 years) and segment 2 after the ‘change-point’ (> 2.69 years). Quantile regression was performed on each segment of the data, per ROI and metric. This regression step generated three parameters about the fitting performed in each segment from which we made some biological inferences. The parameters and their biological inferences are detailed below:

Intercept 1 = The estimated value at birth (age = 0) derived from a regression model of metrics already normalized to the young adult template.

Slope 1 = The rate of change in normalized metric values per year for segment 1, inferred as rate of growth until the ‘change-point’ age, expressed in percentage points relative to the young adult reference.

Slope 2 = The rate of change in normalized metric values per year for segment 2, inferred as rate of growth after the ‘change-point’ age, expressed in percentage points relative to the young adult reference.

## 3 Results

The statistical analysis in Section 2.8 described how the whole brain volume normalized (Δ%) data points were partitioned into two segments. The results from the non-parametric quantile regression analysis performed on each of those segments at the whole brain, total WM, and the grouped pathways (commissural, associative, projections and cerebellar pathways) are presented in Tables 1, 2 for global measurements, and Tables 3, 4 for regional measurements. The individual ROI-wise measurements are provided in the supplement for reference (see Supplementary Tables S1 and S2).

**Table 1:** The intercept values of the global measurements obtained from the quantile regression performed on Segment 1 (< 2.69 years).

		Global intercept values of measurements from Segment 1				
Summary statistics	Tissue	Volume (%)	Fractional anisotropy (%)	Mean diffusivity (%)	Radial diffusivity (%)	Axial diffusivity (%)
Mean	White matter	32	63	134	166	110
Median	White matter	32	65	134	158	112
Standard deviation	White matter	11	10	13	29	8
Maximum	White matter	53	80	158	250	123
Minimum	White matter	12	45	112	122	92
Affine Scaling	Whole brain	45				

Intercept 1 shows the relative percentage of volume, anisotropy (FA), and diffusivity (MD, RD, and AD) at birth with reference to the young adult brain (100%). The whole brain affine scaling or referred in the paper as whole brain volume measurement, is a constant across the entire brain and is included as a reference in the last row of the table.

**Table 2:** The slopes of the global measurements obtained from the quantile regression performed on Segment 1 (< 2.69 years) and Segment 2 (> 2.69 years).

		Global slope values of measurements from Segment 1 and Segment 2									
Summary statistics	Tissue	Volume (Δ%/year)		Fractional anisotropy (Δ%/year)		Mean diffusivity (*Δ*%/year)		Radial diffusivity (Δ%/year)		Axial diffusivity (Δ%/year)	
		Segments		Segments		Segments		Segments		Segments	
		1	2	1	2	1	2	1	2	1	2
Mean	White matter	14.4	1.5	12.5	0.5	−15.2	−0.4	−27.2	−0.7	−6.3	−0.2
Median	White matter	13.9	1.3	11.7	0.4	−15.6	−0.4	−26.4	−0.7	−7.1	−0.2
Standard Deviation	White matter	6.1	1.0	4.0	0.3	8.1	0.2	17.3	0.4	5.0	0.2
Maximum	White matter	29.9	4.1	21.4	1.3	2.8	−0.2	2.2	−0.1	4.3	0.5
Mininum	White matter	3.4	−0.2	3.0	0.0	−31.9	−0.7	−83.9	−1.6	−14.8	−0.4
Affine Scaling	Whole brain	19.9	0.1								

The slope from Segment 1 corresponds to the yearly rate of percentage change in the volume and DTI measurements from birth to 2.69 years, while the slope from Segment 2 corresponds to the yearly rate of percentage change beyond 2.69 years until early adulthood. The whole brain affine scaling is a constant across the entire brain and therefore, only one value is shown for each segment (1 and 2), respectively. Keys: Δ%/year = percentage change in metric per year.

**Table 3:** The intercept values averaged for each WM ROI of the pathway group shown in the first column.

Pathway	Pathway-averaged intercept values for Segment 1				
	Volume (%)	Fractional anisotropy (%)	Mean diffusivity (%)	Radial diffusivity (%)	Axial diffusivity (%)
Commissural	24	66	141	200	115
Association	36	60	137	163	114
Projection	29	63	131	169	106
Cerebellar	30	68	122	143	103

The values represent the average measurement at birth with respect to the young adult brain.

**Table 4:** The slope values averaged for each WM ROI comprising the pathways shown in the first column.

Pathway	Pathway-averaged Slope values for Segment 1 and Segment 2									
	Volume (Δ%/year)		Fractional Anisotropy (Δ%/year)		Mean diffusivity (Δ%/year)		Radial diffusivity (Δ%/year)		Axial diffusivity (Δ%/year)	
	Segment		Segment		Segment		Segment		Segment	
	1	2	1	2	1	2	1	2	1	2
Commissural	20.8	0.8	16.6	0.2	−21.3	−0.4	−51.3	−0.6	−7.8	−0.3
Association	15.7	1.2	12.3	0.5	−16.9	−0.4	−25.7	−0.7	−8.6	−0.2
Projection	8.7	2.4	11.1	0.5	−13.2	−0.4	−25.5	−0.9	−5.0	−0.1
Cerebellar	16.4	1.2	10.8	0.4	−8.7	−0.4	−15.9	−0.6	−1.3	−0.2

Slope 1 represents the average relative rate of growth of a measurement to reach adult brain values, for Segment 1 (< 2.69 years of age). Slope 2 represents the average relative rate of growth of a measurement to reach adult brain values, for the region evaluated, in the years following the first 2.69 years (Segment 2).

### 3.1 Global volumetric and DTI measurements

Tables 1, 2 show the intercept (Segment 1) and slope values (Segments 1 and 2), respectively for all global volume and DTI measurements. For the rest of the manuscript, the intercept corresponding to Segment 1 will be referred to as ‘Intercept 1’ and the slopes corresponding to Segments 1 and 2 will be referred to as ‘Slope 1’ and ‘Slope 2’, respectively. All values reported in the tables are normalized with respect to the median value observed in the developed young-adult brain template, which is defined as 100%. Therefore, the regression intercept reflects the estimated value for a metric at birth (i.e age = 0), derived from a regression model of metrics already normalized to the adult template. For example, an intercept with a value of 40% indicates that the metric at birth is 40% of the young adult reference value, whereas an intercept of 140% indicates a value that is 40% greater than the adult reference value. The slopes represent the rate of change in normalized metric values per year, expressed in percentage points relative to the adult reference value.

In the context of the current analysis, for example, at birth, (Intercept 1, at age = 0 years) the whole brain volume is 45% (last row, Table 1) of the young adult brain volume, indicating that the brain of children younger than 3 years old have only developed 45% of the reference young-adult brain volume. The volume changes at a rapid rate of 20% per year (Slope 1, last row, Table 1) until 2.69 years, thereafter, considerably slows down in growth to 0.1% per year (Slope 2, last row, Table 2) to reach the adult brain size.

Furthermore, at birth, the global WM is 32% (Table 1, row 2) of the volume in young-adults. In comparison, WM anisotropy (FA) is 65% of the adult FA values (Table 1, row 2), while diffusivity (MD, RD and AD) values are at 134, 158 and 112% of the young-adult WM diffusivity values, respectively (Table 1, row 2).

In the first few years following birth, Slope 1 for global WM volume and FA are comparable at 13.9 and 11.7% per year, respectively (Table 2, row 2). It indicates that WM volume and its maturation are progressing at a similar rate, albeit at a slightly slower trajectory compared to the rapidly increasing whole brain volume. It should be noted that while MD changes by −15.6% per year, (Table 2, row 2), it is comparable to the rate of increase in FA in these initial years. RD decreases slightly more rapidly at −26.4% per year to arrive at adult brain RD values. The change in AD is noticeably slower in comparison at −7.1% per year (Table 2).

The rate of change considerably slows down for all metrics after 2.69 years, as seen from Slope 2 values in Table 2. In general, both anisotropy (FA = 0.4% per year) and diffusivity (MD = −0.4%, AD = 0.2% and RD = 0.7% per year) rates go down significantly. Interestingly, while the global brain volume can be observed to change at a very slow rate of 0.1% per year (last row), the change in WM volume was still noticeable at a yearly rate of 1.3% (row 2) to reach the adult brain size.

### 3.2 Regional volumetric and DTI measurements

For the purpose of simplicity and clarity, here we present results from ‘pathway-averaged’ statistical estimates. For each pathway group, the intercept 1, slope 1 and slope 2 parameters are averaged and presented in Tables 3, 4. Please note, we also refer to some relevant estimates of specific ROIs. However, all ROI-wise estimates are tabulated in Supplementary Tables S1and S2 in the supplement for reference. Considering all intercept 1 values from specific ROIs in Supplementary Table S1, we observe that there is a high variability in magnitude of the relative volume change across the WM pathways. When grouped under specific pathway categories, they do not show any noticeable behavior across metrics (Table 3). However, while the differences are not large among Commissural, Association, and Cerebellar pathways for average slope 1 in the first 2.69 years, Commissural pathways on average have a steeper rate of change at 20.8% per year, (Table 4, row 1) compared to the Projection pathways changing 8.7% per year (Table 4, row 3). In addition, on average, the projection pathways have an average higher yearly rate of growth (slope 2 = 2.4% per year) compared to the slower yearly rate of growth of the Commissural pathways (slope 2 = 0.8% per year).

**Figure 2:**
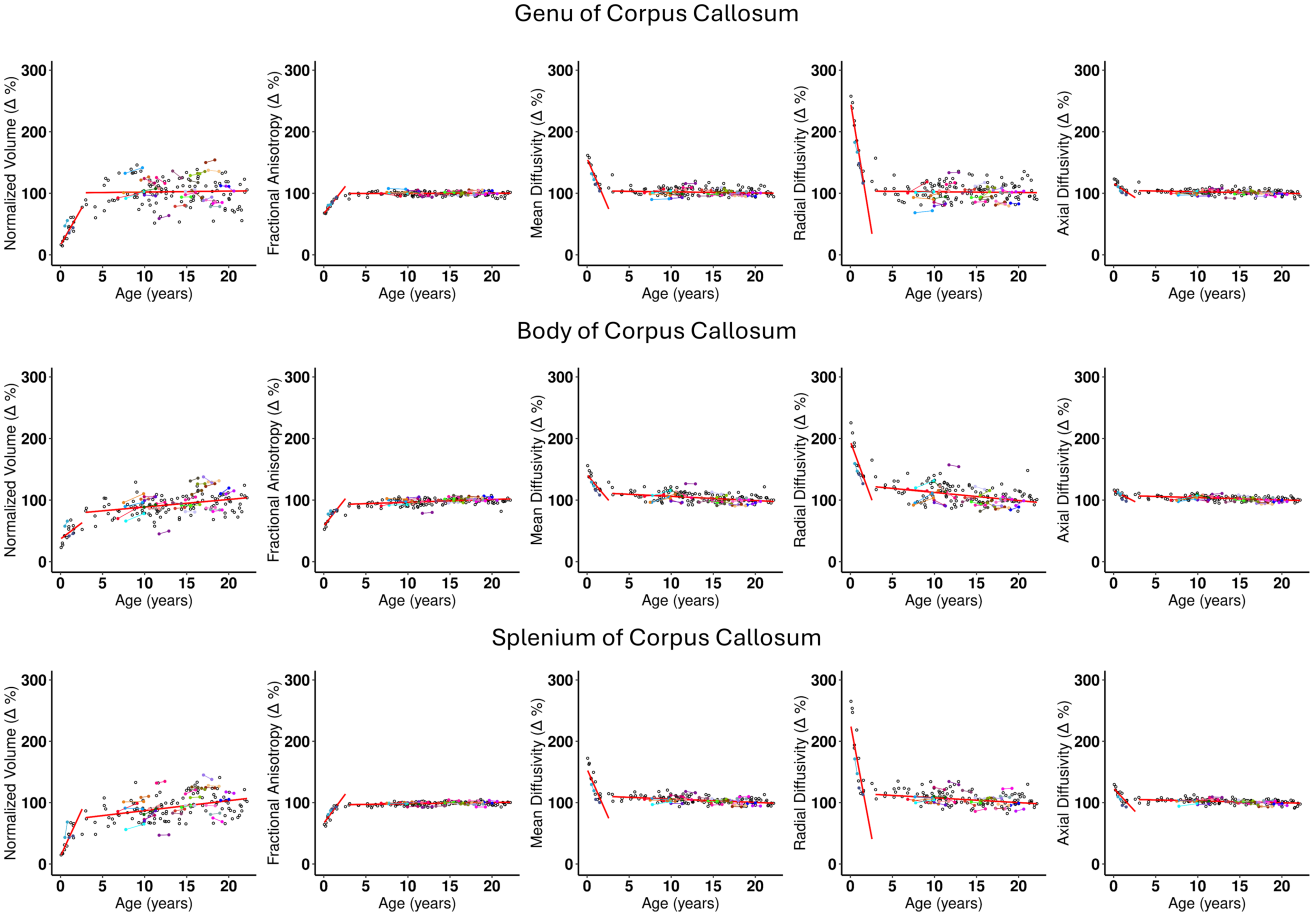
The scatterplots for the three regions of the Corpus Callosum, namely, Genu, Body, and Splenium. Each subject is represented by a dot in the plot. Subjects with longitudinal visits are shown with dots in color with a line connecting the two dots. The x axis is the age distribution in years and the y axis is the normalized relative measurement with respect to the adult brain values, where the values for the adult brain is represented by 100%. The columns from left to right are Normalized (Δ) for volume, Fractional anisotropy, Mean Diffusivity, Radial Diffusivity, and Axial Diffusivity. The two broken red lines in the plot are added after performing a nonparametric linear regression following the partition of the data into two groups around the ‘change-point’ age (as detailed in S.2 section of the supplement). Slope of the first segment is Slope 1, and for segment 2 is Slope 2. These scatter plots show graphically the information tabulated in Table 1. Some important observations from the plots are as follows: (1) Slope 1 is similar for Genu and Splenium and differs from the Slope 1 of the Body. (2) All plots across the DTI metrics, for each region of the corpus callosum show similar behavior of growth trajectory. (3) Slope 1 for RD shows a larger decline for Genu and Splenium compared to the Body. (4) Slope 2 is relatively flat across most of the measurements shown with a few exceptions: Normalized volume (Δ) for Splenium, and RD (Δ) for the same region shows a slightly larger rate of change compared to other metrics and regions. This indicates that the splenium continues to grow in volume in early childhood at a slightly faster rate to achieve adult brain volume, in comparison to almost no growth in the Genu or a slight increase in volume in the Body of the Corpus Callosum. (5) At individual level, the few subjects with longitudinal data follows the general trend of the overall developmental growth trajectory.

**Figure 3:**
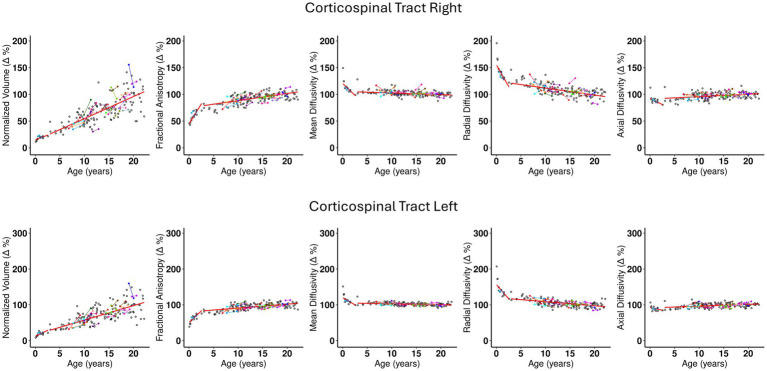
The scatterplots for Corticospinal tract- Right and Left. The description of the plot’s characteristics are the same as explained in caption for Figure 2. Important observations from these plots are: (1) The left and right structures show the similar behavior with regards to distribution of points and the trajectory of the fitted line. (2) Slope 1 for the normalized volume (Δ%) is less steep than Slope 1 of normalized FA (Δ%). (3) There is a steep decrease in RD values as seen from Slope 1 compared to other DTI metrics. (4) MD (Δ%) does not show much change in the initial couple years and is relatively flat thereafter. (5) AD is flat almost across all ages. (6) Slope 2 for volume continues to be steep and does not show a slow down even after 21 years. (7) While significantly less steep than volume, FA, RD, and MD show a moderate increase and decrease, in Slope 1, respectively. (8) Slope 2 for FA appears to be increasing slightly, and this is complimented by a similar trajectory of decrease in RD. Slope 2 for MD appears to be flat. (9) At individual level, the few subjects with longitudinal data follows the general trend of the overall developmental growth trajectory.

The Corticospinal tract and Corpus Callosum, belonging to the Commissural and Projection pathways respectively, show distinct behavior for intercept 1, slopes 1 and 2 (see Supplementary Tables S1 and S2). The Corticospinal tract and Genu and Splenium of Corpus Callosum have similar intercept 1 values approximately ranging between 12 and 16% of the adult brain volume. However, a distinction can be observed at their yearly rate of change. For example, slope 1 of the Corticospinal tract indicates a comparatively slower rate of change of less than 7% per year, (see Supplementary Table S2, rows 5 and 6) compared to the Corpus Callosum, where the Genu and Splenium change at a similar rate of 23.9–29.9% per year, respectively (Supplementary Table S2, rows 1 and 3). Interestingly, the body of the Corpus Callosum shows a rate of change of 10.3% per year, which is close to that of the Corticospinal tract. The plots in Figures 2, 3 show the distinct behavior of the growth trajectories for the two pathways. Please refer to section Supplementary Figures S.5A–P of the supplement for plots presented on remaining WM ROIs not shown here.

#### 3.2.1 Deep gray matter

For Putamen and Thalamus, the magnitude of relative volume change is slightly higher than the whole brain. Given the slope 1 and 2 values (see Supplementary Table S2) for these structures are also similar to the whole brain measurements, it confirms that these structures are growing with and at a similar rate as the whole brain. Please refer to Supplementary Figures S.5O–P for plots presented for Putamen and Thalamus regions.

## 4 Discussion

From diffusion MRI data acquired in a cohort of typically developing children, we have characterized the growth trajectories for volume and DTI metrics in several WM pathways and in the Thalamus and Putamen. In all WM structures investigated, the magnitude of the volume increase from birth to young adulthood was much larger than the magnitude of the change in any of the DTI metrics.

Volume and DTI metrics share similar shapes of their developmental trajectory with larger changes occurring in the younger years and a tendency to level off approaching young adulthood, in agreement with previous studies (13, 22, 25, 28, 29). However, we found that the slopes of the initial and late phases are quite different between volume and DTI metrics. In general, we found that volume has a more protracted growth in late years when DTI metrics, and FA in particular, appear to have plateaued. There are a few studies that have evaluated volume changes in development including data of the first 2 years. A few among them (22, 28) have evaluated some of the same white matter pathways as the ones evaluated in our study. For the Corticospinal tract, a study referenced in (22) used DTI tractography to estimate volume and is concordant with our results. However, in that study the Corpus Callosum was not investigated. Another study referenced in (28), where they perform volume measurements using tractography, show a rapid increase in volume in the initial years. However, in both Corpus Callosum and Corticospinal tract, their results show a plateau effect at about 10 years, while in ours the Corticospinal tract shows protracted volume growth extending to young adulthood and the Corpus Callosum shows a much earlier plateau at about 3 years. These discrepancies should be further investigated.

Overall, we find that the magnitude of the relative volume at birth compared to young adulthood, and its developmental trajectory, show higher interregional variability compared to that revealed by DTI metrics. This large range of differences in volume growth trajectories across pathways have not been previously systematically investigated and reported. It represents an interesting feature to be further investigated in studies relating structure and function in development. It could be argued that variability may just represent noise and not be representative of true biological variability. However, we find quite consistent trajectories in the left and right tracts of bilateral pathways. Moreover, tracts that are part of the same anatomo-functional pathway also show a similar growth trajectory. For example, the volume growth trajectory of the pontine crossing tract is very similar to that of the Middle Cerebellar Peduncle. This should be expected because axons of the pontine crossing fibers give origin to the Middle Cerebellar Peduncle, although the two tracts show location and architectural features that are very different. On the contrary, the Corticospinal tract, that at the level of the Pons runs adjacent to the pontine crossing fibers, shows a completely different developmental trajectory.

One additional observation is that the magnitude of the volume growth for virtually all WM pathways are larger than that of the whole brain. In fact, the relative whole brain volume that we estimated at birth is 45% of adult volume, indicates that from birth to young adulthood the whole brain approximately doubles in volume. The median relative volume for WM matter pathways at birth was 32%, indicating that WM volume overall triples from birth to young adulthood, with some structures, like the Corticospinal tract and Corpus Callosum that show a 6 fold increase. On the contrary the two deep gray matter structures we examined, Putamen and Thalamus, show findings similar to those of the whole brain.

### 4.1 Biological interpretation of our findings

Although MRI is clearly sensitive to compositional, structural, and architectural changes occurring in brain tissue during development, it is much more difficult to establish the specificity of different MRI derived metrics to distinctly occuring biological processes. Moreover, a complete picture of the postnatal changes occurring in the brain is still not available even from anatomical post-mortem studies in humans and studies with invasive methods in animals. There is a general agreement that the number of long range axonal fibers would not increase after birth (if anything it may decrease due to selective pruning) (44), therefore the increase in volume should not be attributed to an increase in the number of fibers. However, during postnatal development, the overall axon diameter increases both for an increase in the cross-section of the axon itself and for the deposition of additional myelin wraps (45). In the immature brain, there is a higher content of water in the interstitial (inter-axonal) space with respect to the adult brain, resulting in larger inter-axonal distances and lower extra-axonal tortuosity (46). As the brain matures, the water content of the interstitial space decreases (46), which in theory should contribute to a decrease of the local volume of the fiber we measure with DTBM. The volumetric changes that we measure with DTBM are probably the result of these two opposite mechanisms: growth of axon diameter and thickness of myelin sheet, with a shrinkage of the extracellular space. These phenomena also affect the diffusion tensor metrics, although in a different way. It is not completely clear how myelination affects diffusion anisotropy. It is clear that unmyelinated fibers show diffusional anisotropy (46), although myelination may contribute to increased anisotropy by reducing the rate of exchange between intra- and extra-axonal water. For sure, as also demonstrated by methods other than MRI, there is an increased tortuosity of the interstitial space which increases anisotropy, with a reduction of radial diffusivity, and consequently a reduction of mean diffusivity in WM (46).

### 4.2 Strengths and limitations of our study

One strength of our study is that we were able to examine ages ranging from infancy to early adulthood. Imaging the brain at a very young age is challenging due to limitations posed by recruitment, retainment, and data quality due to motion artifacts. This large age range has allowed us to normalize age specific measurements to the values reached in young adulthood. Another strength is the novelty of the methodology used for volumetric measurements and the ability to compare and contrast volumetric and DTI metrics in each region. The DTI-based diffeomorphic registration has allowed visually reliable coregistration of structures across different subjects over a large age range. However, we cannot rule out regional differences in the quality of the registration, resulting in inaccurate information in some areas. For example, we did not include cortical areas in our analysis because, given the high interindividual variability in cortical folding, we were not confident that the intersubject registration would have been meaningful in the cortex. There are a few weaknesses with the study sample we have used for the analysis. We have performed the analysis on mostly cross-sectional data that were acquired on two different scanners. The cross-sectional design is not ideal to evaluate developmental trajectories and can only describe average developmental trends. The added effect of developmental changes with the small sample size for the study precluded the test for scanner-related variability on the results. In addition, the small number of subjects under the age of 2.7 years made it challenging to examine the rapid growth changes at smaller intervals within this period. Also, the relative scarcity of female subjects in the lower age range was not suitable for examination of potential sex related differences in developmental trajectories. Future studies should consider conducting similar analysis with DTBM in a larger sample with longitudinal data.

## 5 Conclusion

DTBM has shown developmental trajectories for WM volume in the human brain that are pathway specific and distinct from those obtained for DTI metrics. In future studies, DTBM should be performed in larger cohorts to assess correlation with cognitive and behavioral changes as well as establishing ranges for normative values.

## Data Availability

The original contributions presented in the study are included in the article/Supplementary material, further inquiries can be directed to the corresponding author.
