# Supplementary material for: Volume changes in white matter pathways from infancy to early adulthood measured using diffusion tensor based morphometry
Source: Front Neurol. 2025 Sep 12;16:1624779. doi: 10.3389/fneur.2025.1624779 (PMC12463617; doi:10.3389/fneur.2025.1624779)
Supplement: Supplementary file 1 [file Data_Sheet_1.pdf]

S.1 Age group distribution

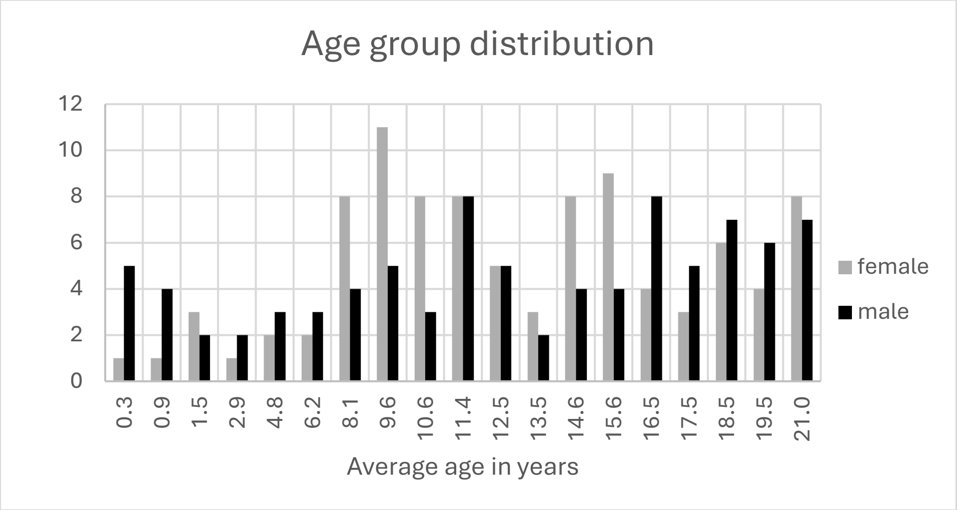

Figure S.1: shows the distribution of age groups for the study. The subjects included in the study were grouped in increments of 6 months before 2 years and thereafter, attempted to group yearly depending on the availability of data. As observed from the graph, there are a smaller number of subjects in the age groups prior to 8 years and thus a few groups were combined between ages 4-7 years. In addition, due to fewer subjects in the younger age group, there are only two female subjects under the age of 1. The number of males and females in the older groups, especially the average age of 18.5-21 years is well balanced and ideal in the creation of the reference template.

S.2: Age group and reference template creation pipeline

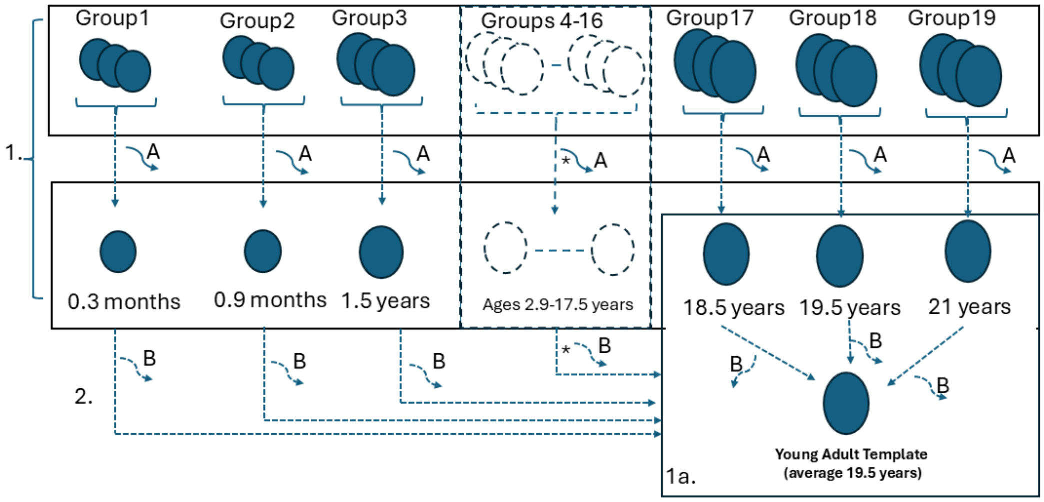

Figure S.2: Shows a graphical representation of the pipeline used to generate the age group and reference templates for the study. The two main steps are categorized as 1 and 2, where in Step1, the individual brains are grouped by age in increments of 6 months in the first two years and yearly increments afterwards, depending on the adequate number of subjects to generate the average template. The first step resulted in 19 age specific templates. In step 1a, the older age groups in the study i.e 18.5 years, 19.5 years, and 21 years average template were used in the creation of the reference template which has the average age of a young adult (19.5 years). Following the generation of the reference template, each group template is registered to the reference template generated from step 1a. It should be noted that in step 1 and 1a, the tensors used in the creation of the templates are re-registered to their respective reference templates to ensure all data across the groups have undergone the same registration effects. The curved arrows indicate the transformation maps generated during the registration of images to their respective reference templates. The transformation maps are combined using the combine-transformations module from TORTOISE software [TORTOISEV4/src/tools/CombineTransformations/combine_transformations.cxx at main · QMICodeBase/TORTOISEV4 · GitHub] to generate a combined transformation that contains information about the overall mapping of individual brains to the young adult template. The transformation combination strategy employed in this work is based on ITK library's MultiTransform class [https://docs.itk.org/projects/doxygen/en/latest/classitk_1_1MultiTransform.html] .

S.3: Anatomical locations of the Region of Interest (ROIs)

(A)

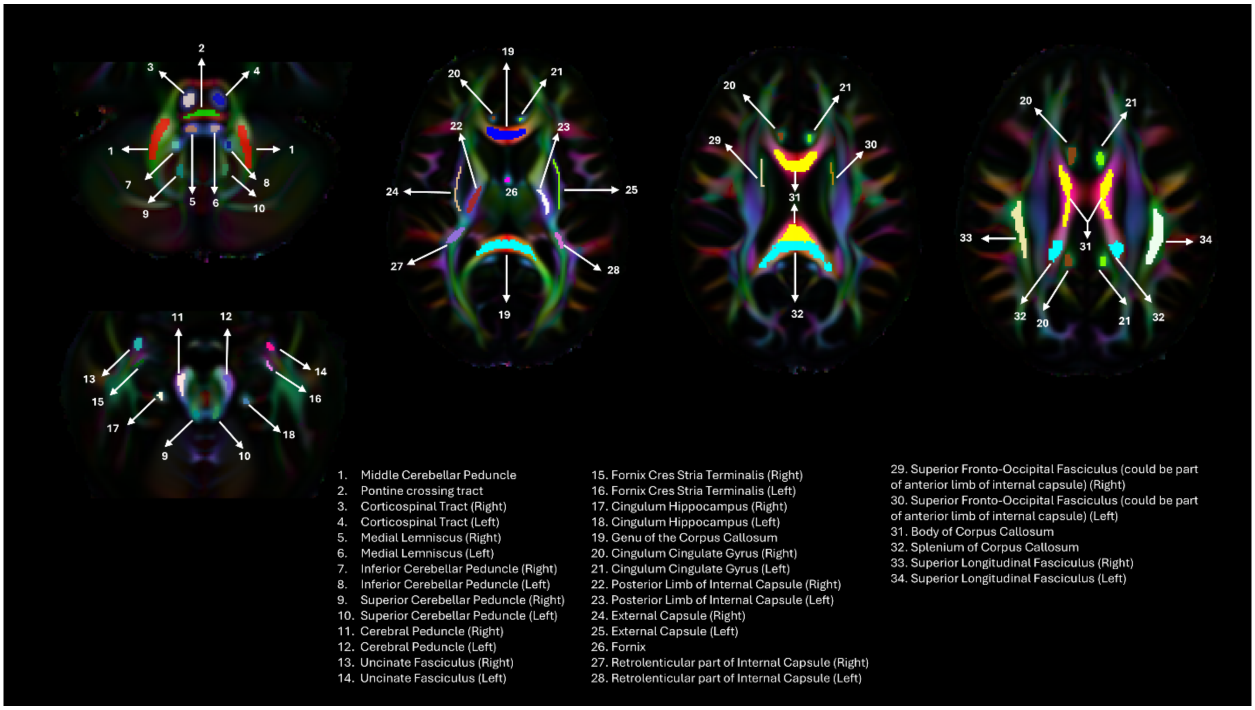

(B)

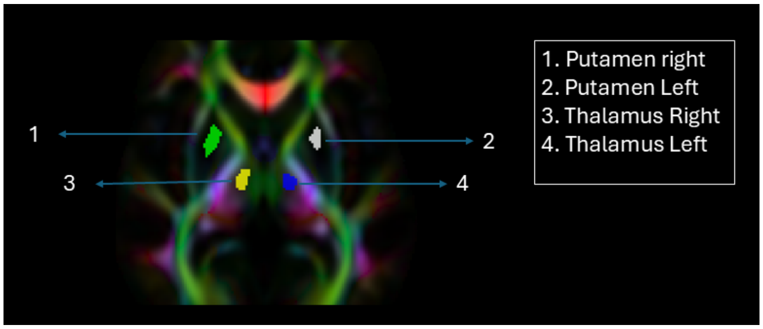

Figure S.3: (A) shows the anatomical locations of the white matter ROIs defined on a Human Connectome (HCP) Directionally Encoded Color (DEC) map. (B) shows the deep gray matter ROIs defined on the young adult template DEC map.

The ROIs used in the study are as follows:

Commissural: Genu, Body, and Splenium of the Corpus Callosum, and Pontine Crossing Tract.

Association: External Capsule, Cingulum Cingulate Gyrus, Cingulum Hippocampus, Fornix Cres Stria Terminalis, Fornix, Superior Longitudinal Fasciculus, Superior Fronto-Occipital Fasciculus, Uncinate Fasciculus.

Projection: Cerebral Peduncle, Posterior Limb of Internal Capsule, Retro-lenticular part of Internal Capsule, Corticospinal Tract, and Medial Lemniscus.

Cerebellar: Inferior, superior, and middle cerebellar peduncle.

Deep gray matter: Putamen and Thalamus

S.4 Statistical analysis

Identifying the Breakpoint Between Infancy and Adulthood

An affine scaling was applied to each individual's whole brain, while being registered to a study specific template. The determinant of this transformation was used to evaluate the age at which infant brains would approximately reach the volume of adult brains. The period after this specific timepoint was also evaluated to verify that the brain volume did

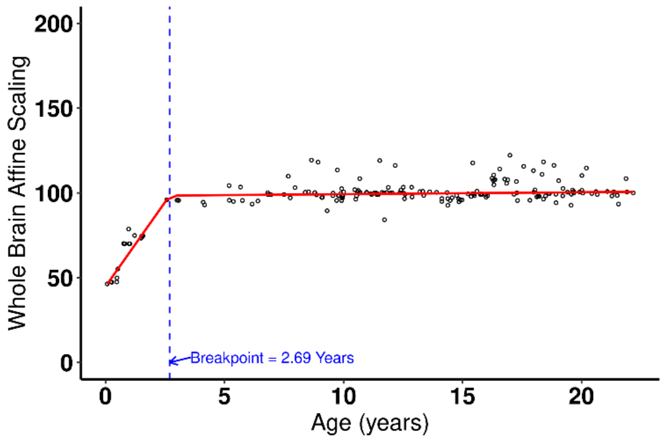

Figure S.4.a: shows the trajectory and breakpoint of the change in whole brain volume between infancy and adulthood using segmented regression. The x-axis shows the ages from early infancy to adulthood (~ 22 years). The y-axis shows the relative percentage change (%) in the whole brain volume with respect to the adult brain. The blue dashed line shows the breakpoint solution reached by the segmented regression algorithm. The red regression lines before (segment1) and after (segment2) the breakpoint, represent the brain volume changes during development vs. early childhood-to-adulthood, respectively.

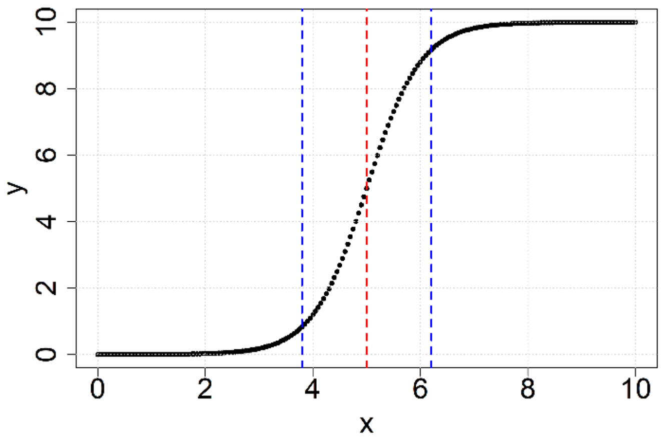

Figure S.4.b: shows an example figure based on arbitrary data to show an inflection point and possible breakpoints. The point where the dashed red line intersects with the curve is the inflection point. Two possible breakpoints, one before (left, dashed blue) and one after (right, dashed blue) the inflection point can be observed, where the asymptote curve tends to plateau.

not change significantly beyond that age. To do this, a 'segmented regression' approach was used.(1, 2) Based on the distribution of the data, we observed that the whole brain volume across age abruptly rose within a few years from infancy and then reached a plateau (see the blank dots in Figure S.4.a). Such trends would typically cause an abrupt 'change-point' in the trajectory, which can be identified as a 'breakpoint' between two linear regressions using the 'segmented' package(1-3) in R.(4) Typically, the 'segmented' package would require one/more tentative breakpoint values to start the iterative process of eventually finding the solutions for each breakpoint. Instead of providing a biased input from our manual observation, we applied a data-driven approach, which provided a meaningful starting point for the 'segmented' algorithm to identify the breakpoint.

The trajectory of the data points across the age in the sample clearly followed a 'curved' plane (see the coordinate points in Figure S.4.a). Such polynomial trends typically contain inflection points, where the second derivative changes signs. Therefore, a breakpoint would typically precede and/or follow an inflection point (see Figure S.4.b). We used this concept to first identify a possible inflection point, using the 'inflection' package(5-7) in R. This was then set as an input to the 'segmented' algorithm to find an accurate breakpoint.

Segment-wise Non-Parametric Regression

Based on the breakpoint computed from the whole brain affine scaling (see Figure S.4.a), all data comprising 'regional' (ROIs) volumes and DTI metrics were separated into two segments. Segment 1 – comprised all subjects having '$\text{age} \leq \text{breakpoint}_{\text{whole-brain-affine-scaling}}$' and Segment 2 – comprised all data having '$\text{age} > \text{breakpoint}_{\text{whole-brain-affine-scaling}}$'. Non-parametric linear regressions were performed on each of these sections independently for each ROI across all metrics. Non-parametric approaches are less prone to outliers and extreme values in the data and therefore provide more robust estimates of the slope (e.g., rate of change volume per year) and intercept (e.g., volume at birth, i.e. age = 0) of each segment. To do this, we applied the quantile regression(8, 9) approach using the *'quantreg'* package in R. Note that the segmented regression performed for the whole brain affine scaling (Figure S.4.a) also incorporated the same non-parametric approach.

We did not perform the segmented regression at the ROI-level for several reasons. First, understandably, we do not have sufficient data at the early infancy and childhood age. Therefore, the segmented regression can be prone to generating variable breakpoints even within bilateral ROIs due to sample variations in the early development years. Secondly, we wanted to investigate how the local volume and white matter compositional properties change with respect to the global changes in volume. Therefore, keeping the break point

fixed, by virtue of the distribution in the global volume allow for accurate comparison across modalities. Therefore, we performed quantile regression for the 50th percentile (τ = 0.5, i.e. the median) within each segment. The individual regression lines were then plotted within each respective segment to showcase the difference in effect of brain growth between early development (Segment 1) and childhood-to-adulthood (Segment 2) years in the same figure. Essentially, the same principle of segmented regression applies, excepting it was done by keeping the breakpoint fixed across all ROIs and MRI-derived metrics.

Table S1 The intercept values of the ROI-wise measurements obtained from the quantile regression performed on Segment 1 (< 2.69 years).

		**Regional Intercept Values of Measurements from Segment 1**				
**Region of Interest**	**Tissue**	**Volume (%)**	**Fractional Anisotropy (%)**	**Mean Diffusivity (%)**	**Radial Diffusivity (%)**	**Axial Diffusivity (%)**
**Genu Of Corpus Callosum**	White Matter	16	67	157	250	116
**Body Of Corpus Callosum**	White Matter	37	58	141	195	115
**Splenium Of Corpus Callosum**	White Matter	13	66	155	229	123
**Pontine Crossing Tract**	White Matter	29	74	113	125	104
**Corticospinal Tract Right**	White Matter	15	45	120	155	93
**Corticospinal Tract Left**	White Matter	12	50	120	157	92
**Medial Lemniscus Right**	White Matter	20	61	127	160	102
**Medial Lemniscus Left**	White Matter	24	64	130	159	102
**External Capsule Right**	White Matter	53	66	133	150	118
**External Capsule Left**	White Matter	52	66	133	154	118
**Cingulum Cingulate Gyrus Right**	White Matter	33	48	148	180	112

**Cingulum Cingulate Gyrus Left**	White Matter	27	50	144	195	112
**Cingulum Hippocampus Right**	White Matter	48	64	136	145	118
**Cingulum Hippocampus Left**	White Matter	37	54	134	150	119
**Fornix**	White Matter	37	80	112	122	101
**Fornix Cres Stria Terminalis Right**	White Matter	41	78	121	135	105
**Fornix Cres Stria Terminalis Left**	White Matter	33	73	125	141	110
**Superior Longitudinal Fasciculus Right**	White Matter	23	51	158	204	118
**Superior Longitudinal Fasciculus Left**	White Matter	17	51	157	207	120
**Superior Fronto-Occipital Fasciculus Right**	White Matter	31	52	145	172	113
**Superior Fronto-Occipital Fasciculus Left**	White Matter	33	46	142	168	115
**Uncinate Fasciculus Right**	White Matter	38	64	136	157	115
**Uncinate Fasciculus Left**	White Matter	39	62	138	159	113
**Cerebral Peduncle Right**	White Matter	26	66	138	204	107
**Cerebral Peduncle Left**	White Matter	25	65	130	190	105
**Posterior Limb Of Internal Capsule Right**	White Matter	47	73	134	166	112

**Posterior Limb Of Internal Capsule Left**	White Matter	49	76	132	156	113
**Retrolenticular Part Of Internal Capsule Right**	White Matter	32	70	141	172	119
**Retrolenticular Part Of Internal Capsule Left**	White Matter	39	64	138	175	117
**Middle Cerebellar Peduncle**	White Matter	28	74	132	156	114
**Inferior Cerebellar Peduncle Right**	White Matter	27	58	123	146	99
**Inferior Cerebellar Peduncle Left**	White Matter	33	65	120	137	101
**Superior Cerebellar Peduncle Right**	White Matter	30	70	122	142	104
**Superior Cerebellar Peduncle Left**	White Matter	34	72	115	131	97
**Putamen Right**	Gray Matter	47	79	128	129	126
**Putamen Left**	Gray Matter	40	72	127	129	124
**Thalamus Right**	Gray Matter	61	66	125	129	114
**Thalamus Left**	Gray Matter	69	70	125	131	116

Values for 34 white matter (WM) and 4 gray matter (GM) ROIs are shown.

Table S2 The slopes of the ROI-wise measurements obtained from the quantile regression performed on Segment 1 (< 2.69 years) and Segment 2 (> 2.69 years).

		**Regional Slope Values of Measurements from Segment 1 and Segment 2**									
**Region of Interest**	**Tissue**	**Volume (Δ%/year)**		**Fractional Anisotropy (Δ%/year)**		**Mean Diffusivity (Δ%/year)**		**Radial Diffusivity (Δ%/year)**		**Axial Diffusivity (Δ%/year)**	
		Segment		Segment		Segment		Segment		Segment	
		1	2	1	2	1	2	1	2	1	2
**Genu Of Corpus Callosum**	White Matter	23.9	0.2	17.4	0.0	-31.9	-0.2	-83.9	-0.1	-9.0	-0.2
**Body Of Corpus Callosum**	White Matter	10.3	1.2	17.3	0.5	-16.2	-0.7	-37.1	-1.3	-7.0	-0.4
**Splenium Of Corpus Callosum**	White Matter	29.9	1.6	18.5	0.2	-31.2	-0.6	-73.4	-0.8	-14.8	-0.3
**Pontine Crossing Tract**	White Matter	19.0	0.0	13.1	0.1	-6.0	-0.3	-10.8	-0.3	-0.6	-0.4
**Corticospinal Tract Right**	White Matter	3.4	4.1	14.9	1.3	-9.0	-0.3	-16.2	-1.3	-4.9	0.4
**Corticospinal Tract Left**	White Matter	6.7	4.0	13.1	1.1	-8.0	-0.3	-15.8	-1.2	-3.0	0.5
**Medial Lemniscus Right**	White Matter	13.3	2.1	13.8	0.6	-12.4	-0.6	-26.5	-0.9	-1.5	-0.2
**Medial Lemniscus Left**	White Matter	12.5	2.1	7.7	0.6	-13.2	-0.5	-17.2	-1.0	-1.1	-0.3
**External Capsule Right**	White Matter	13.7	0.7	11.7	0.4	-16.7	-0.3	-23.2	-0.5	-11.1	-0.1
**External Capsule Left**	White Matter	16.6	0.8	11.2	0.5	-15.3	-0.3	-26.4	-0.5	-10.7	-0.1
**Cingulum Cingulate Gyrus Right**	White Matter	6.6	2.3	11.6	1.1	-21.6	-0.6	-28.7	-1.3	-11.1	0.1
**Cingulum Cingulate Gyrus Left**	White Matter	9.0	2.6	15.1	1.1	-19.0	-0.6	-42.0	-1.2	-9.8	0.0

**Cingulum Hippocampus Right**	White Matter	10.7	1.5	7.2	0.6	-15.6	-0.5	-16.2	-0.6	-12.3	-0.3
**Cingulum Hippocampus Left**	White Matter	18.8	1.0	11.0	1.0	-15.3	-0.6	-17.6	-1.0	-12.0	-0.3
**Fornix**	White Matter	15.9	1.2	3.0	0.3	2.8	-0.6	2.2	-0.8	2.3	-0.4
**Fornix Cres Stria Terminalis Right**	White Matter	11.3	1.2	6.9	0.4	-7.5	-0.4	-11.8	-0.4	-3.3	-0.2
**Fornix Cres Stria Terminalis Left**	White Matter	16.3	1.3	7.5	0.3	-12.3	-0.2	-14.5	-0.4	-7.2	-0.2
**Superior Longitudinal Fasciculus Right**	White Matter	14.1	2.1	16.0	0.4	-29.4	-0.4	-47.6	-0.7	-12.5	-0.2
**Superior Longitudinal Fasciculus Left**	White Matter	20.6	2.4	17.5	0.4	-29.2	-0.5	-49.3	-0.9	-14.0	-0.1
**Superior Fronto-Occipital Fasciculus Right**	White Matter	25.1	0.4	17.8	0.3	-20.5	-0.3	-31.3	-0.5	-3.8	-0.2
**Superior Fronto-Occipital Fasciculus Left**	White Matter	23.6	0.6	21.4	0.6	-19.7	-0.2	-28.8	-0.6	-8.4	-0.1
**Uncinate Fasciculus Right**	White Matter	16.8	0.5	12.8	0.2	-15.5	-0.4	-24.2	-0.4	-7.7	-0.2
**Uncinate Fasciculus Left**	White Matter	16.3	-0.2	13.7	0.2	-19.4	-0.3	-26.5	-0.3	-8.3	-0.2
**Cerebral Peduncle Right**	White Matter	5.6	2.7	7.6	0.4	-14.0	-0.7	-37.3	-1.4	-4.5	-0.2
**Cerebral Peduncle Left**	White Matter	6.2	3.1	9.8	0.5	-3.0	-0.7	-21.0	-1.6	1.3	-0.4

**Posterior Limb Of Internal Capsule Right**	White Matter	8.4	1.0	11.4	0.1	-17.4	-0.3	-32.6	-0.3	-7.0	-0.2
**Posterior Limb Of Internal Capsule Left**	White Matter	7.1	1.3	11.1	0.0	-16.5	-0.2	-28.2	-0.2	-7.5	-0.3
**Retrolenticular Part Of Internal Capsule Right**	White Matter	12.9	1.6	9.8	0.2	-20.8	-0.3	-29.1	-0.3	-10.5	-0.2
**Retrolenticular Part Of Internal Capsule Left**	White Matter	11.5	1.5	11.7	0.3	-17.9	-0.3	-31.6	-0.6	-10.7	-0.2
**Middle Cerebellar Peduncle**	White Matter	18.5	0.6	13.5	0.1	-16.9	-0.3	-29.7	-0.3	-7.1	-0.2
**Inferior Cerebellar Peduncle Right**	White Matter	18.9	1.6	13.7	0.7	-9.8	-0.4	-19.4	-0.6	-1.3	-0.2
**Inferior Cerebellar Peduncle Left**	White Matter	13.3	1.9	8.2	0.8	-8.3	-0.5	-13.5	-0.7	-3.5	-0.1
**Superior Cerebellar Peduncle Right**	White Matter	16.0	1.0	9.0	0.4	-6.5	-0.5	-11.6	-0.9	1.1	-0.4
**Superior Cerebellar Peduncle Left**	White Matter	15.4	0.6	9.7	0.3	-2.1	-0.5	-5.5	-0.8	4.3	-0.3
**Putamen Right**	Gray Matter	23.2	-0.5	3.3	1.1	-14.5	-0.3	-14.4	-0.3	-14.5	-0.2
**Putamen Left**	Gray Matter	32.4	-0.7	3.1	1.6	-14.2	-0.3	-14.8	-0.4	-13.0	-0.1
**Thalamus Right**	Gray Matter	12.1	0.8	8.3	0.9	-14.8	-0.4	-13.7	-0.6	-10.8	-0.1
**Thalamus Left**	Gray Matter	8.0	0.5	7.2	0.8	-13.0	-0.4	-13.3	-0.6	-12.4	-0.1

S.5 Segmented linear regression plots for WM pathways

(A)

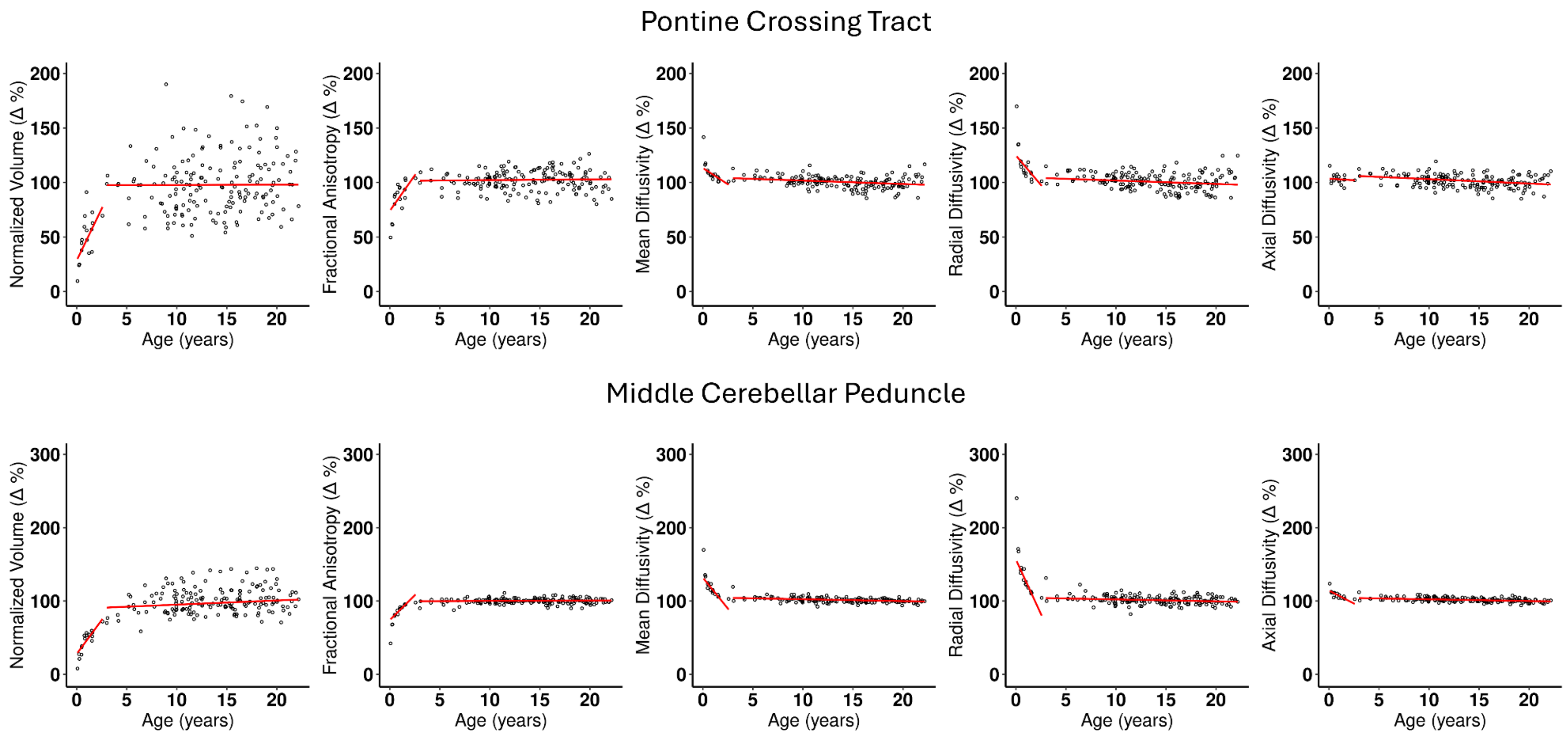

(B)

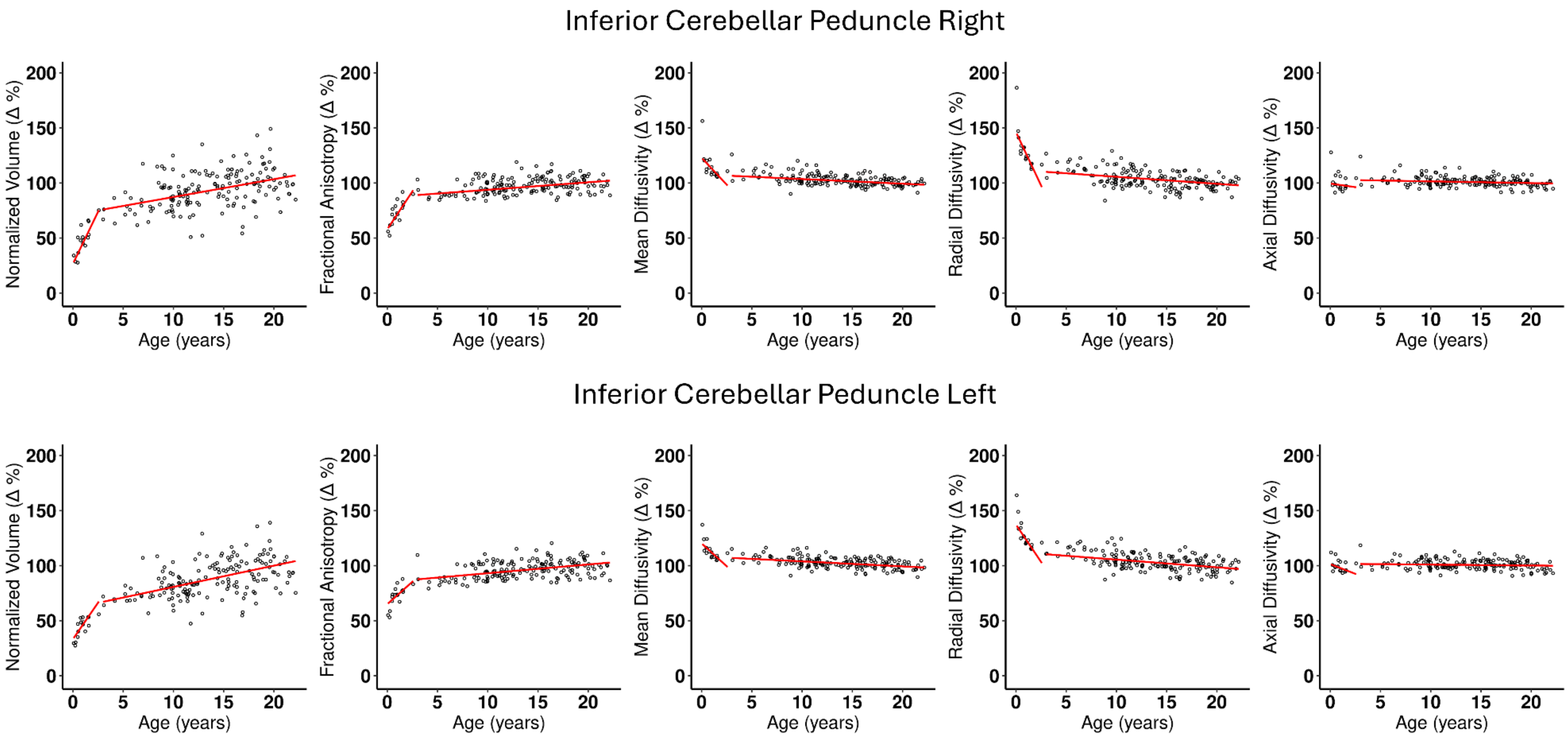
Inferior Cerebellar Peduncle Right
Inferior Cerebellar Peduncle Left
Normalized Volume (Δ %)
Fractional Anisotropy (Δ %)
Mean Diffusivity (Δ %)
Radial Diffusivity (Δ %)
Axial Diffusivity (Δ %)
Age (years)

(C)

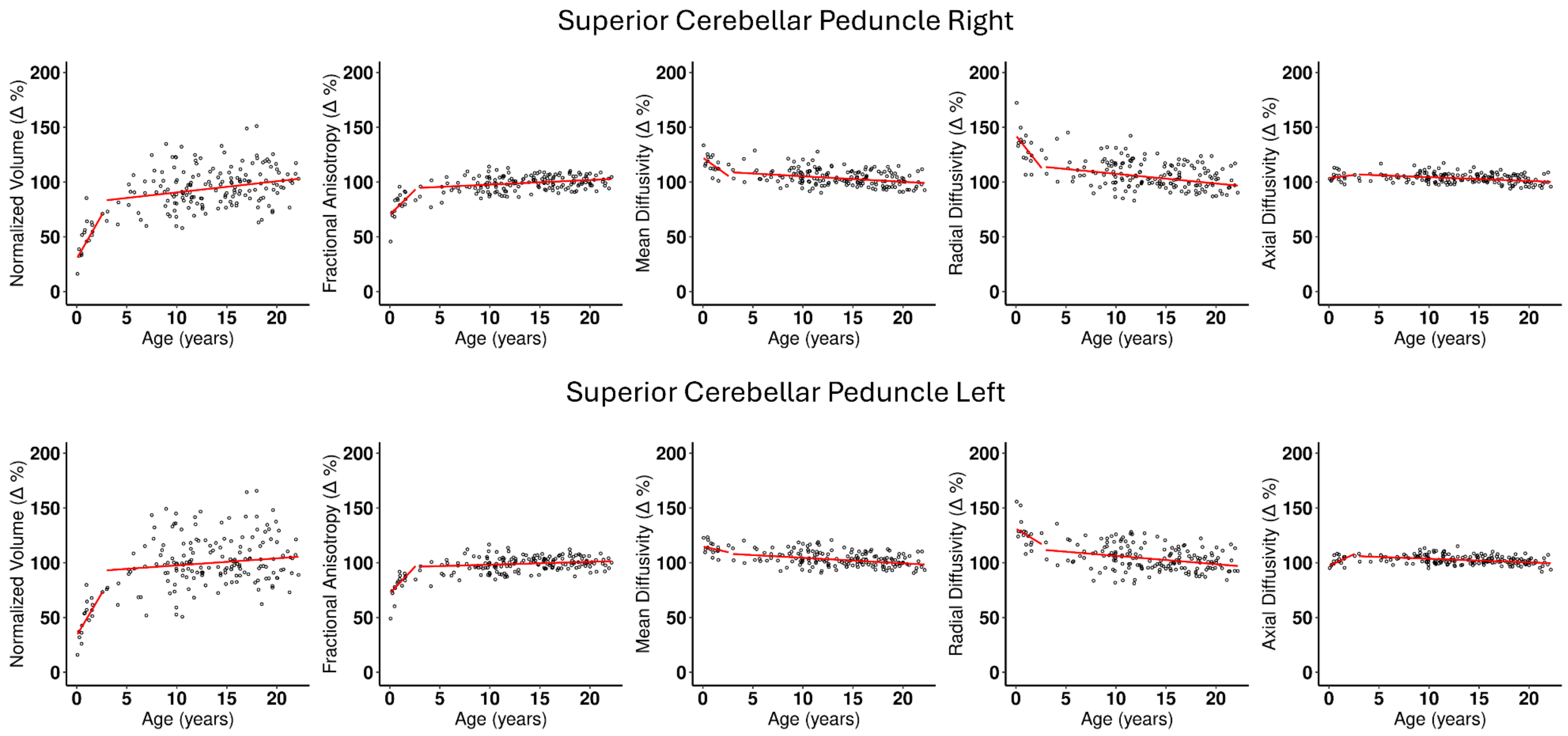
Superior Cerebellar Peduncle Right
Normalized Volume (Δ %)
Fractional Anisotropy (Δ %)
Mean Diffusivity (Δ %)
Radial Diffusivity (Δ %)
Axial Diffusivity (Δ %)
0
50
100
150
200
Age (years)
0
5
10
15
20
Superior Cerebellar Peduncle Left
Normalized Volume (Δ %)
Fractional Anisotropy (Δ %)
Mean Diffusivity (Δ %)
Radial Diffusivity (Δ %)
Axial Diffusivity (Δ %)
Age (years)

(D)

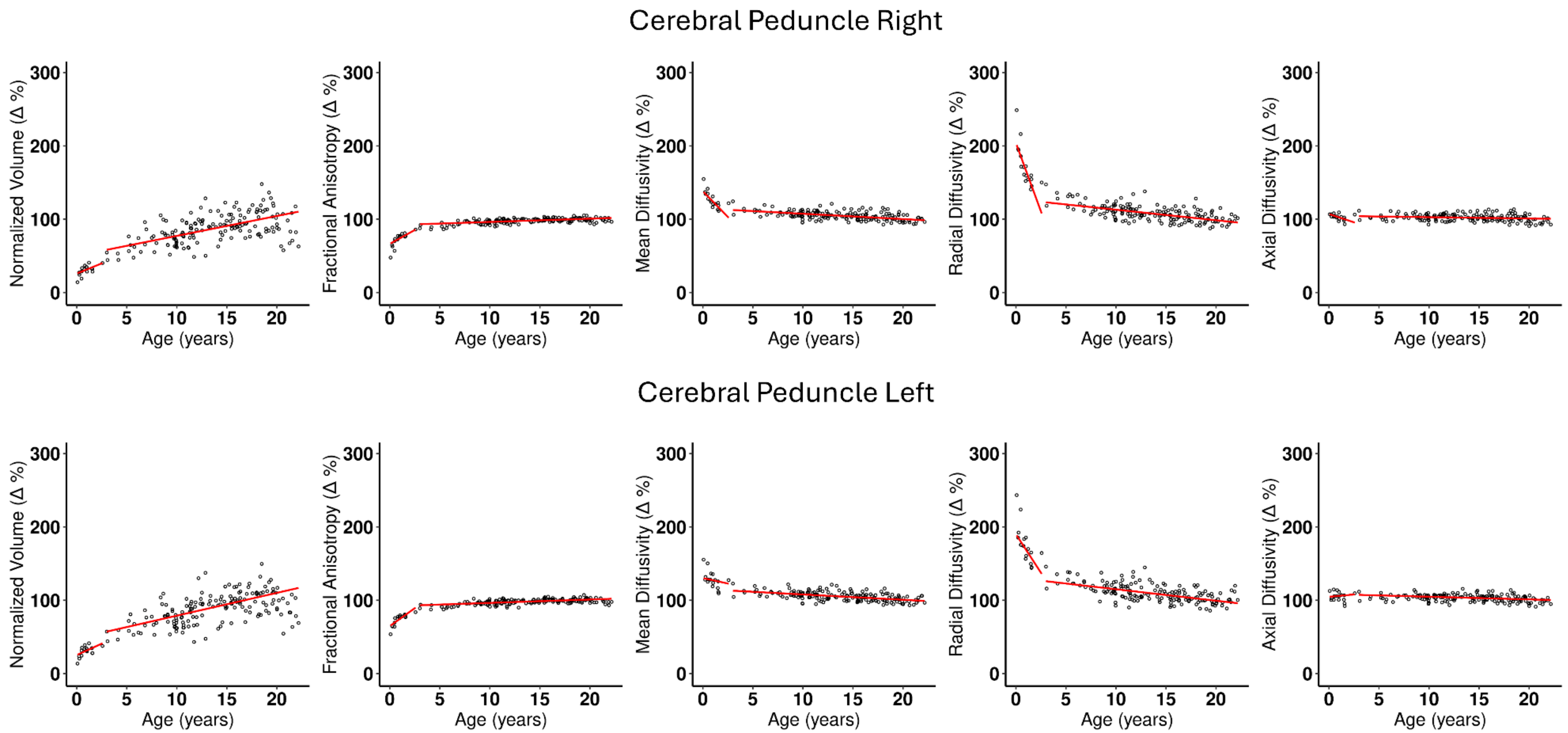
Cerebral Peduncle Right
Cerebral Peduncle Left
Normalized Volume (Δ %)
Fractional Anisotropy (Δ %)
Mean Diffusivity (Δ %)
Radial Diffusivity (Δ %)
Axial Diffusivity (Δ %)
0
100
200
300
Age (years)
0
5
10
15
20

(E)

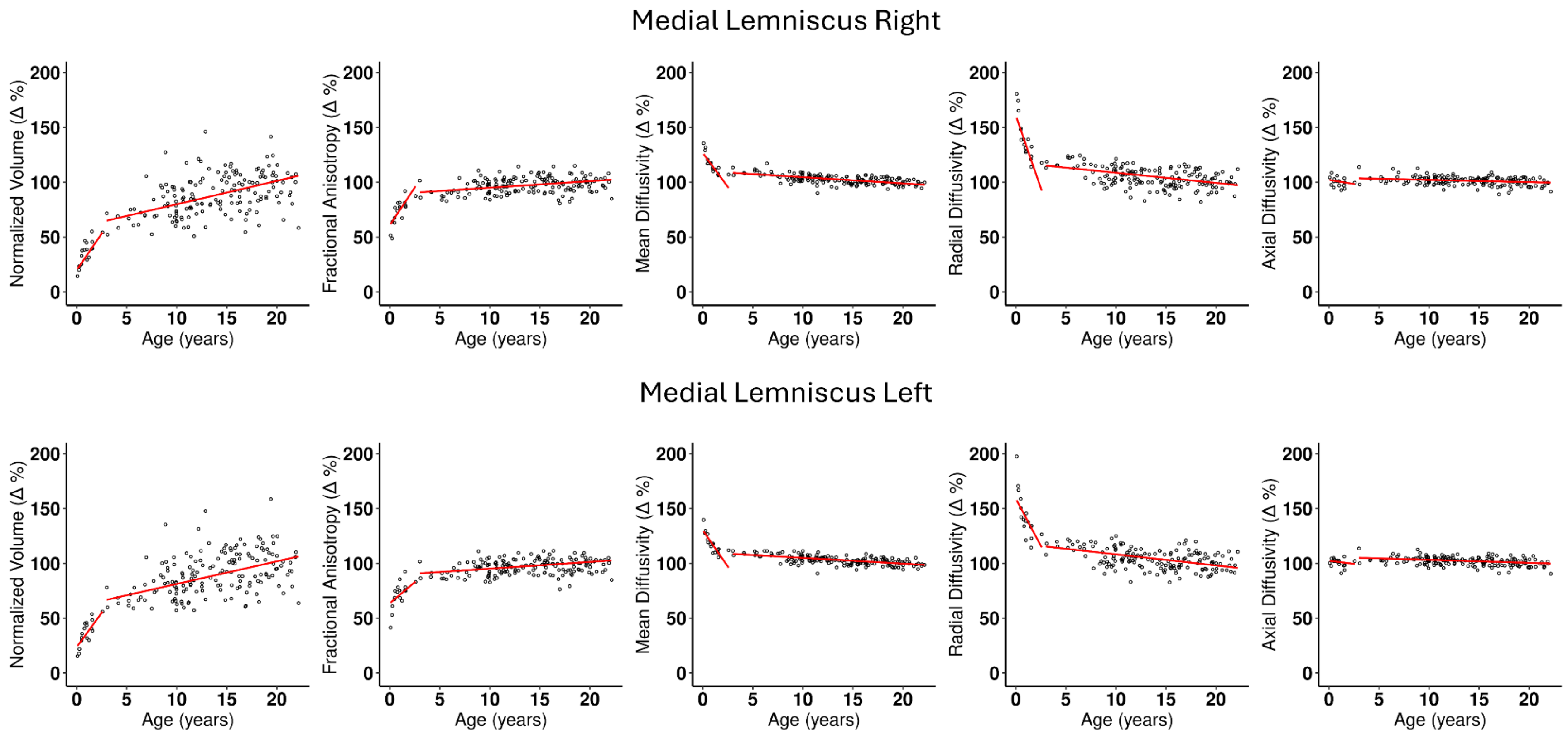
Medial Lemniscus Right
Medial Lemniscus Left
Normalized Volume (Δ %)
Fractional Anisotropy (Δ %)
Mean Diffusivity (Δ %)
Radial Diffusivity (Δ %)
Axial Diffusivity (Δ %)
Age (years)
0
50
100
150
200
5
10
15
20

(F)

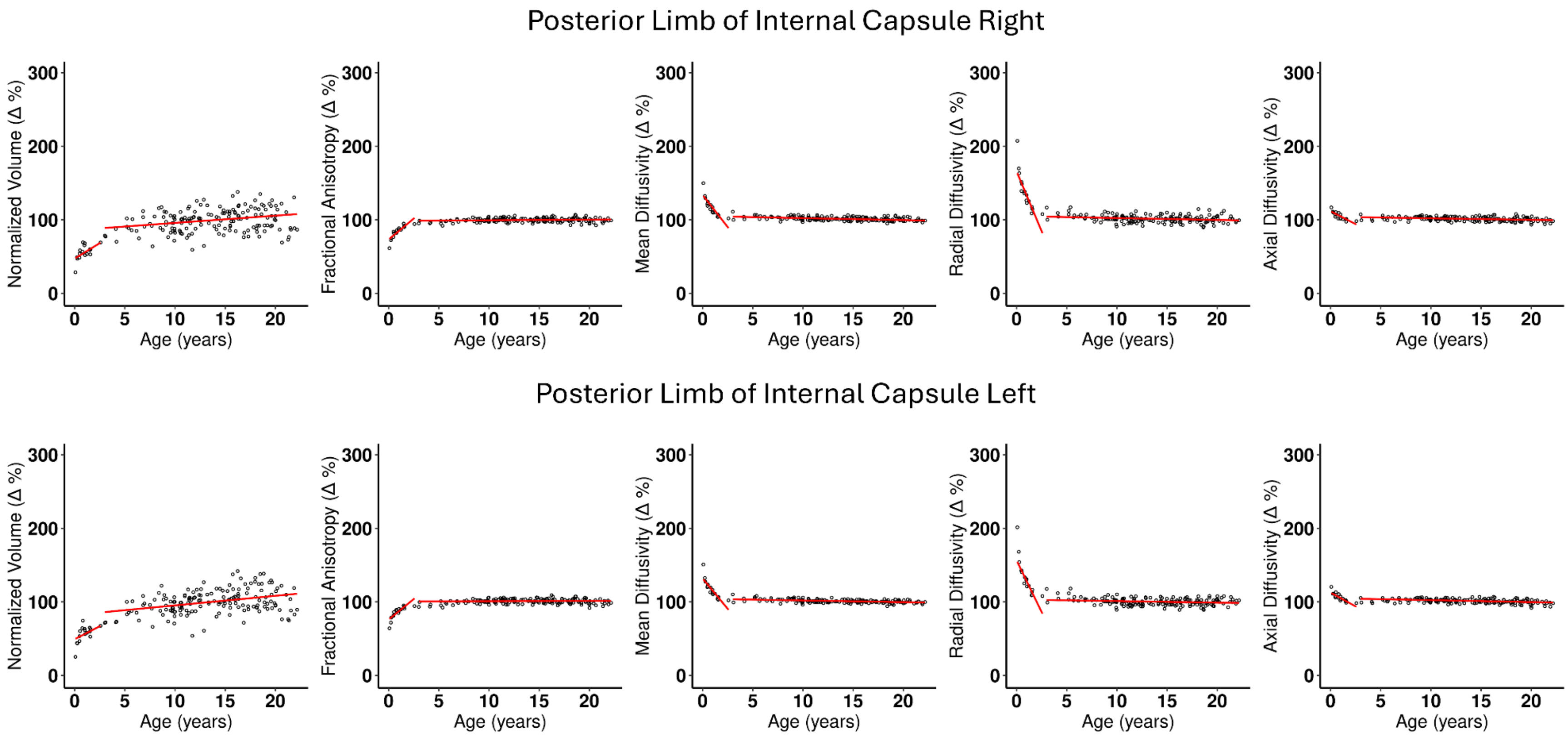
Posterior Limb of Internal Capsule Right
Posterior Limb of Internal Capsule Left
Normalized Volume (Δ %)
Fractional Anisotropy (Δ %)
Mean Diffusivity (Δ %)
Radial Diffusivity (Δ %)
Axial Diffusivity (Δ %)
Age (years)
0
100
200
300
5
10
15
20

(G)

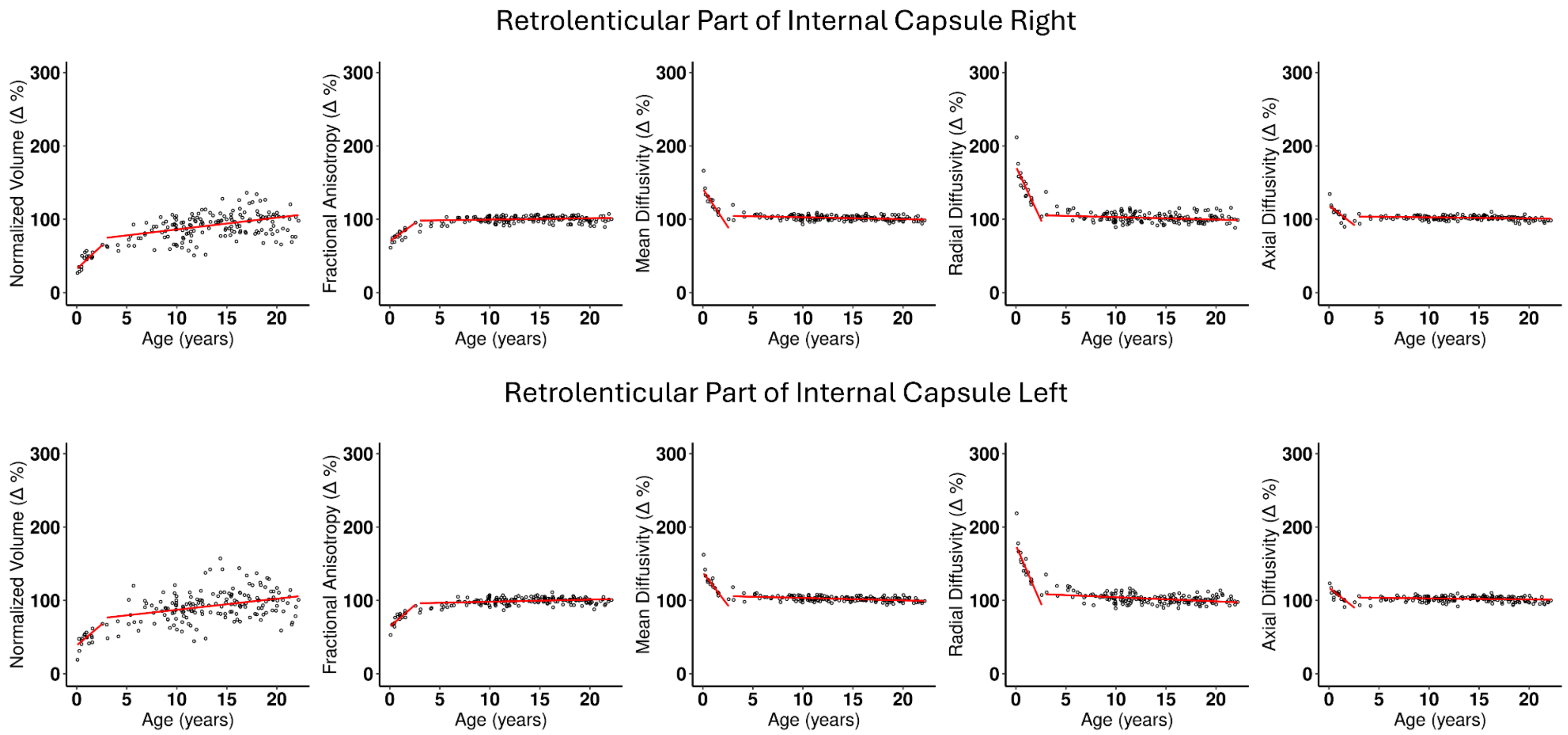
Retrolenticular Part of Internal Capsule Right
Retrolenticular Part of Internal Capsule Left
Normalized Volume (Δ %)
Fractional Anisotropy (Δ %)
Mean Diffusivity (Δ %)
Radial Diffusivity (Δ %)
Axial Diffusivity (Δ %)
Age (years)
0
100
200
300
5
10
15
20

(H)

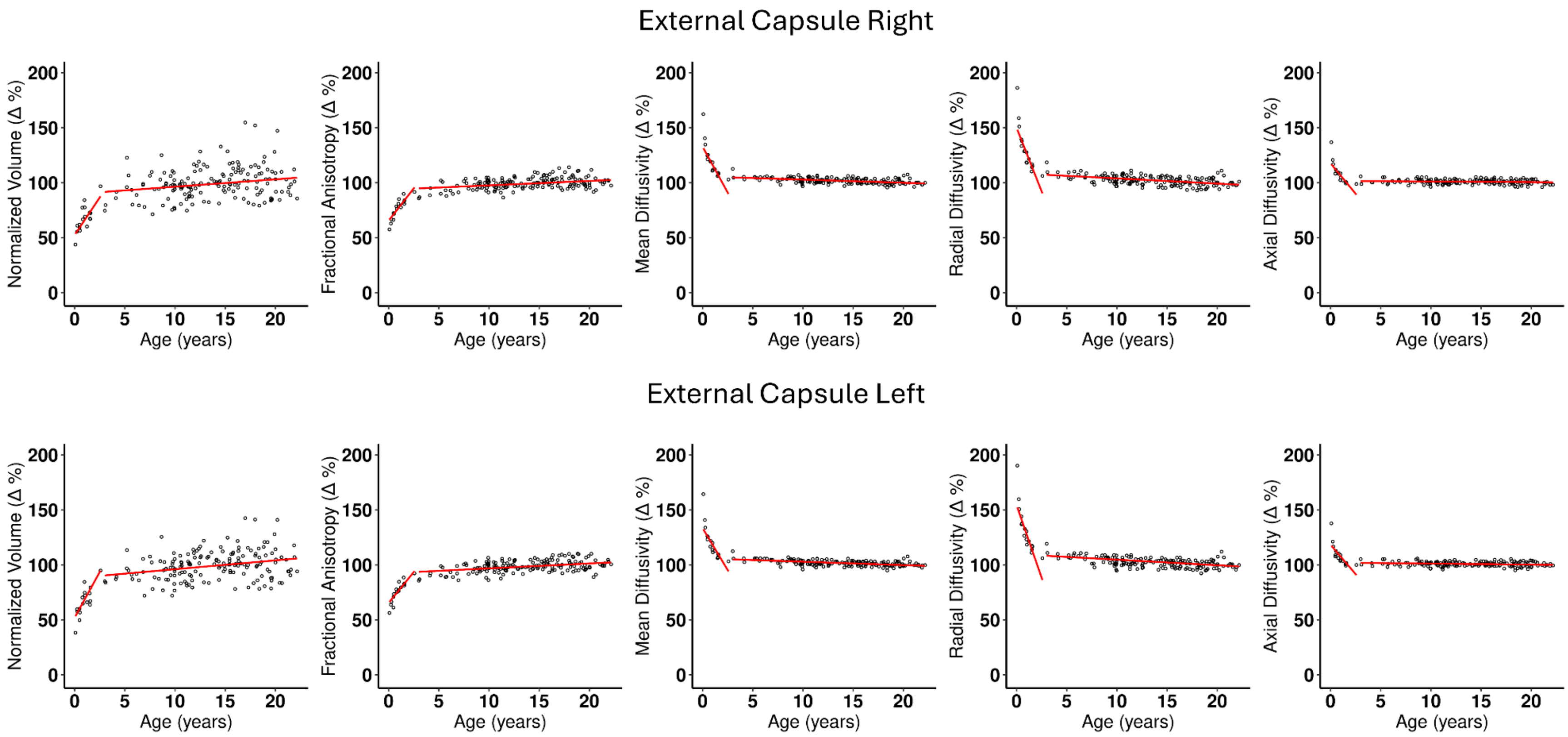
External Capsule Right
Normalized Volume (Δ %)
Fractional Anisotropy (Δ %)
Mean Diffusivity (Δ %)
Radial Diffusivity (Δ %)
Axial Diffusivity (Δ %)
200
150
100
50
0
0
5
10
15
20
Age (years)
External Capsule Left
Normalized Volume (Δ %)
Fractional Anisotropy (Δ %)
Mean Diffusivity (Δ %)
Radial Diffusivity (Δ %)
Axial Diffusivity (Δ %)
Age (years)

(I)

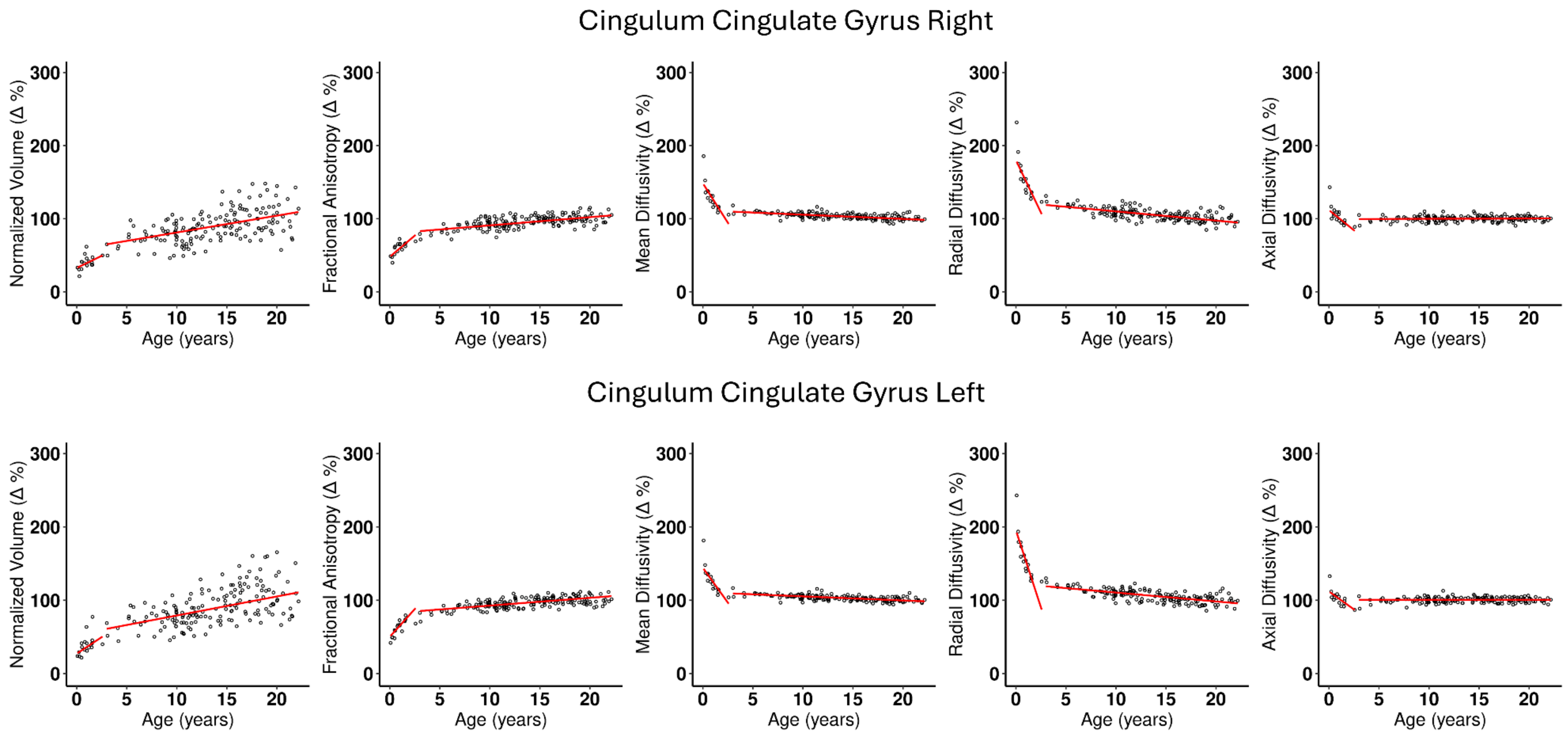
Cingulum Cingulate Gyrus Right
Cingulum Cingulate Gyrus Left
Normalized Volume (Δ %)
Fractional Anisotropy (Δ %)
Mean Diffusivity (Δ %)
Radial Diffusivity (Δ %)
Axial Diffusivity (Δ %)
Age (years)
0
100
200
300
5
10
15
20

(J)

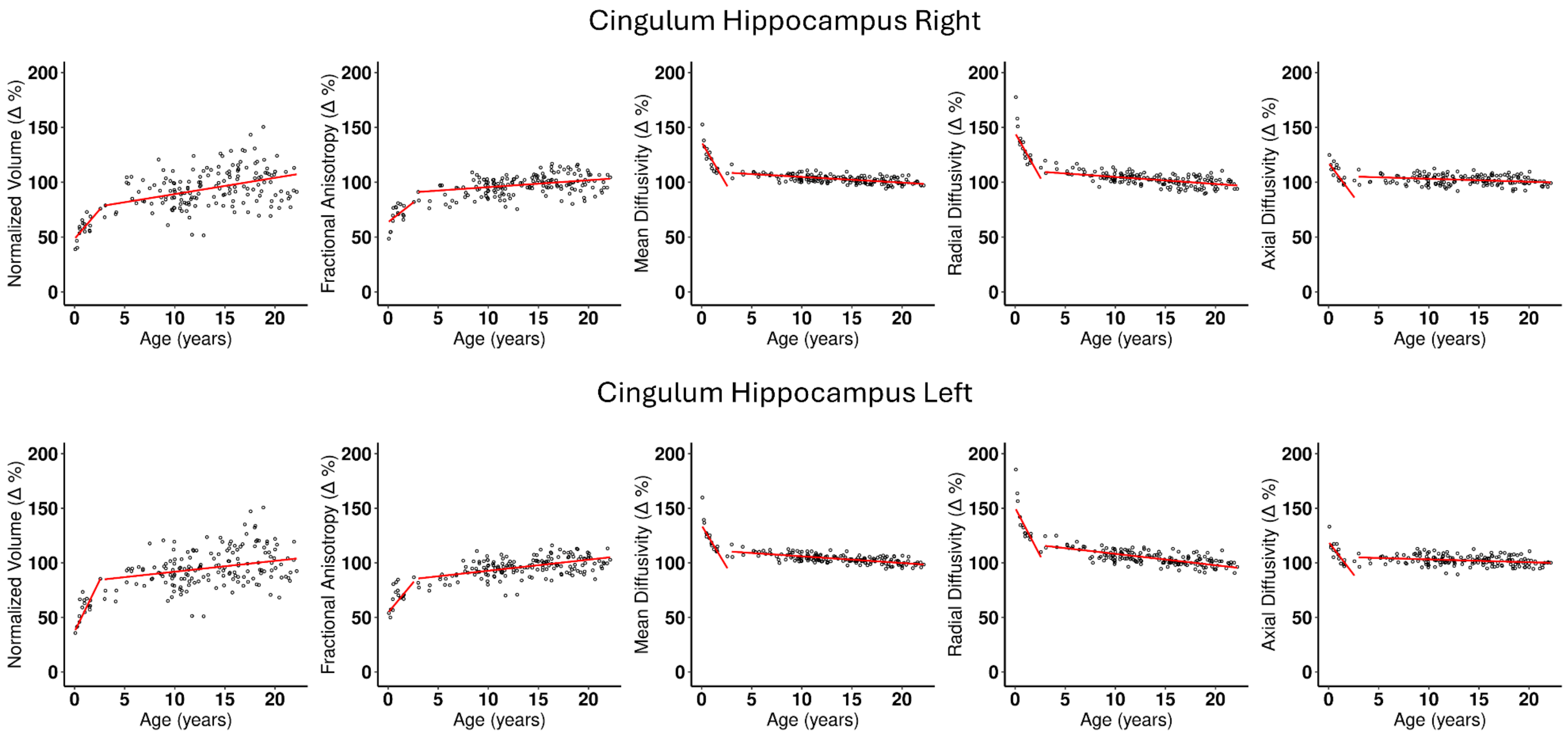
Cingulum Hippocampus Right
Normalized Volume (Δ %)
Fractional Anisotropy (Δ %)
Mean Diffusivity (Δ %)
Radial Diffusivity (Δ %)
Axial Diffusivity (Δ %)
Age (years)
Cingulum Hippocampus Left
Normalized Volume (Δ %)
Fractional Anisotropy (Δ %)
Mean Diffusivity (Δ %)
Radial Diffusivity (Δ %)
Axial Diffusivity (Δ %)
Age (years)

(K)

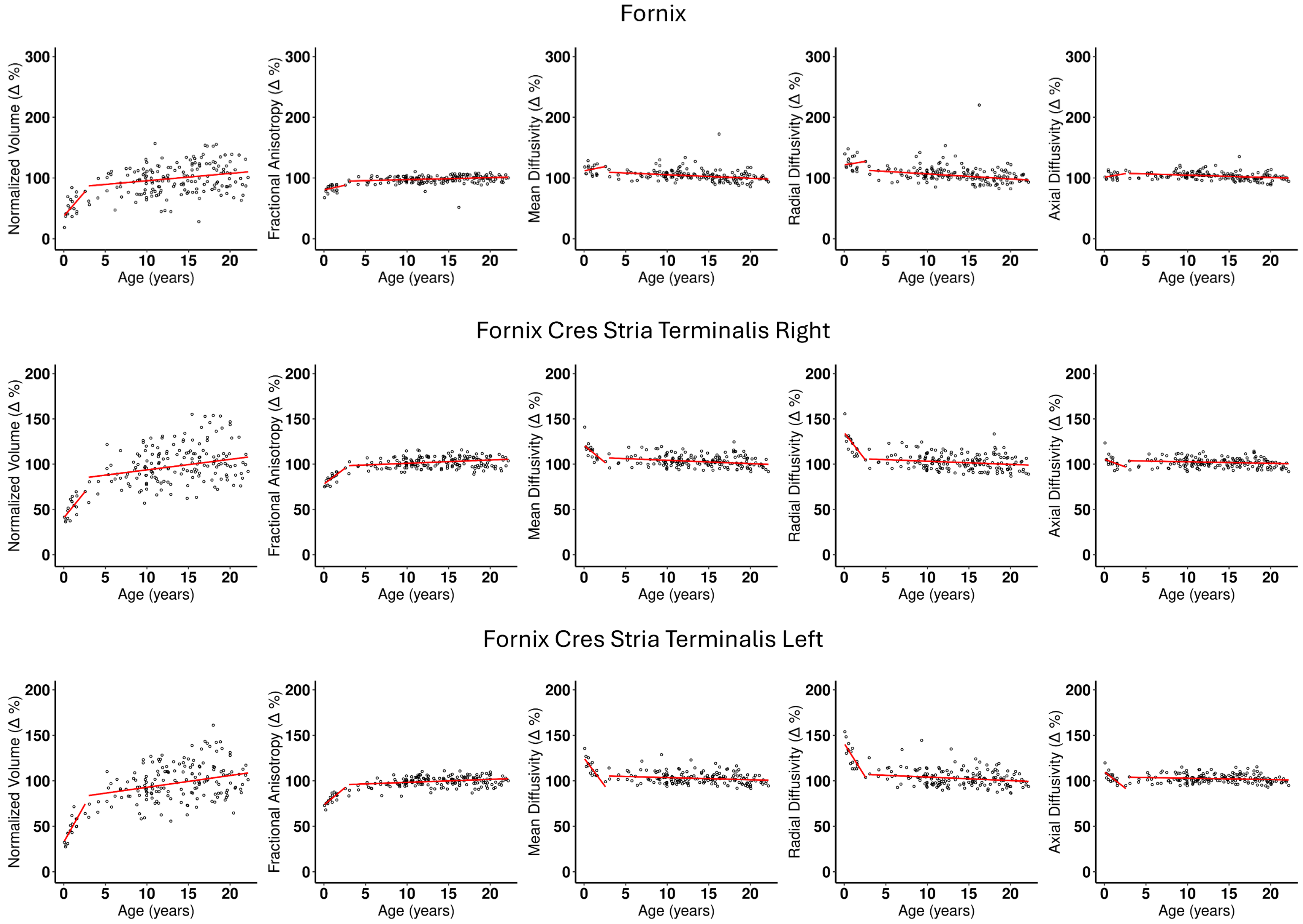

Fornix
Fornix Cres Stria Terminalis Right
Fornix Cres Stria Terminalis Left
Normalized Volume (Δ %)
Fractional Anisotropy (Δ %)
Mean Diffusivity (Δ %)
Radial Diffusivity (Δ %)
Axial Diffusivity (Δ %)
Age (years)

(L)

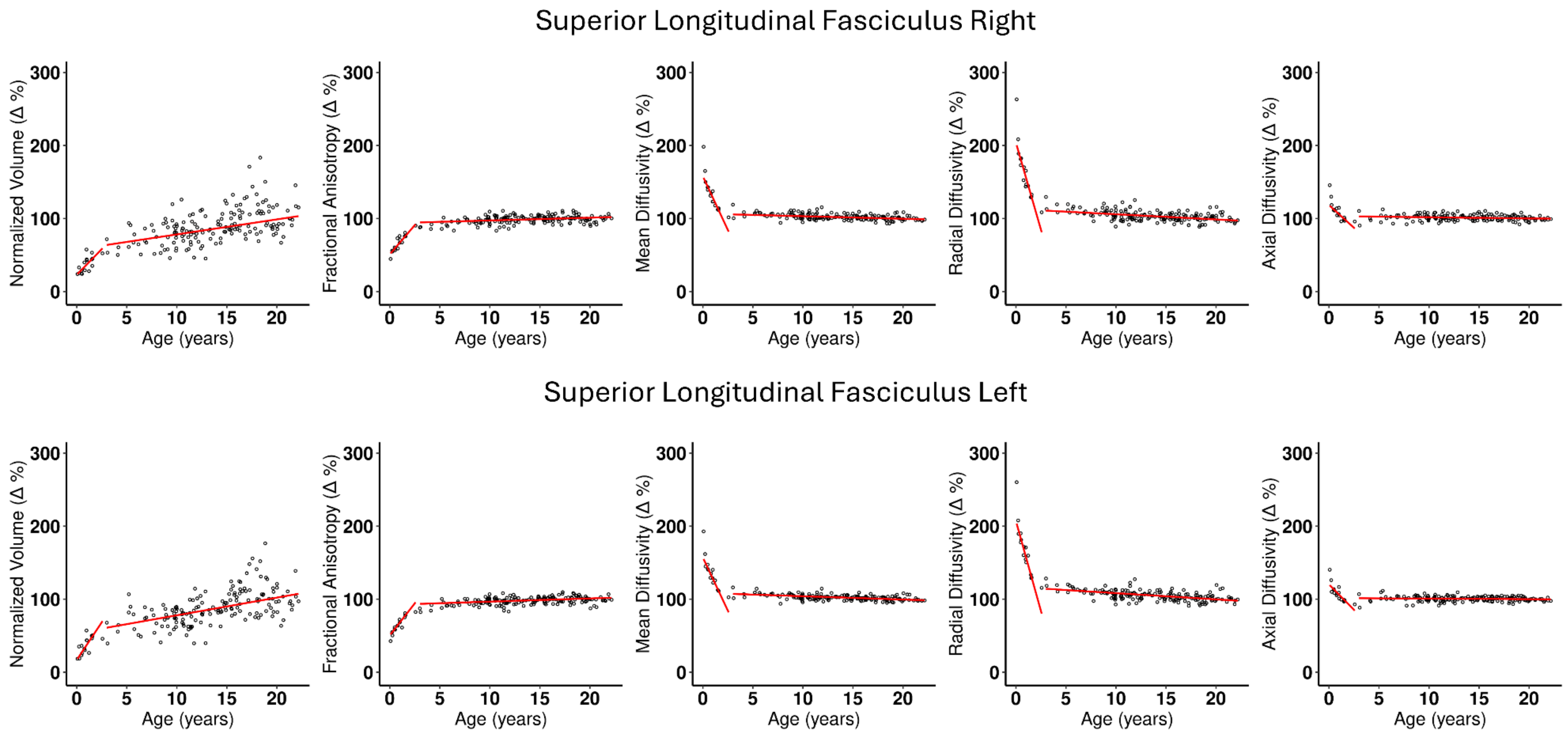
Superior Longitudinal Fasciculus Right
Superior Longitudinal Fasciculus Left
Normalized Volume (Δ %)
Fractional Anisotropy (Δ %)
Mean Diffusivity (Δ %)
Radial Diffusivity (Δ %)
Axial Diffusivity (Δ %)
Age (years)
0
100
200
300
5
10
15
20

(M)

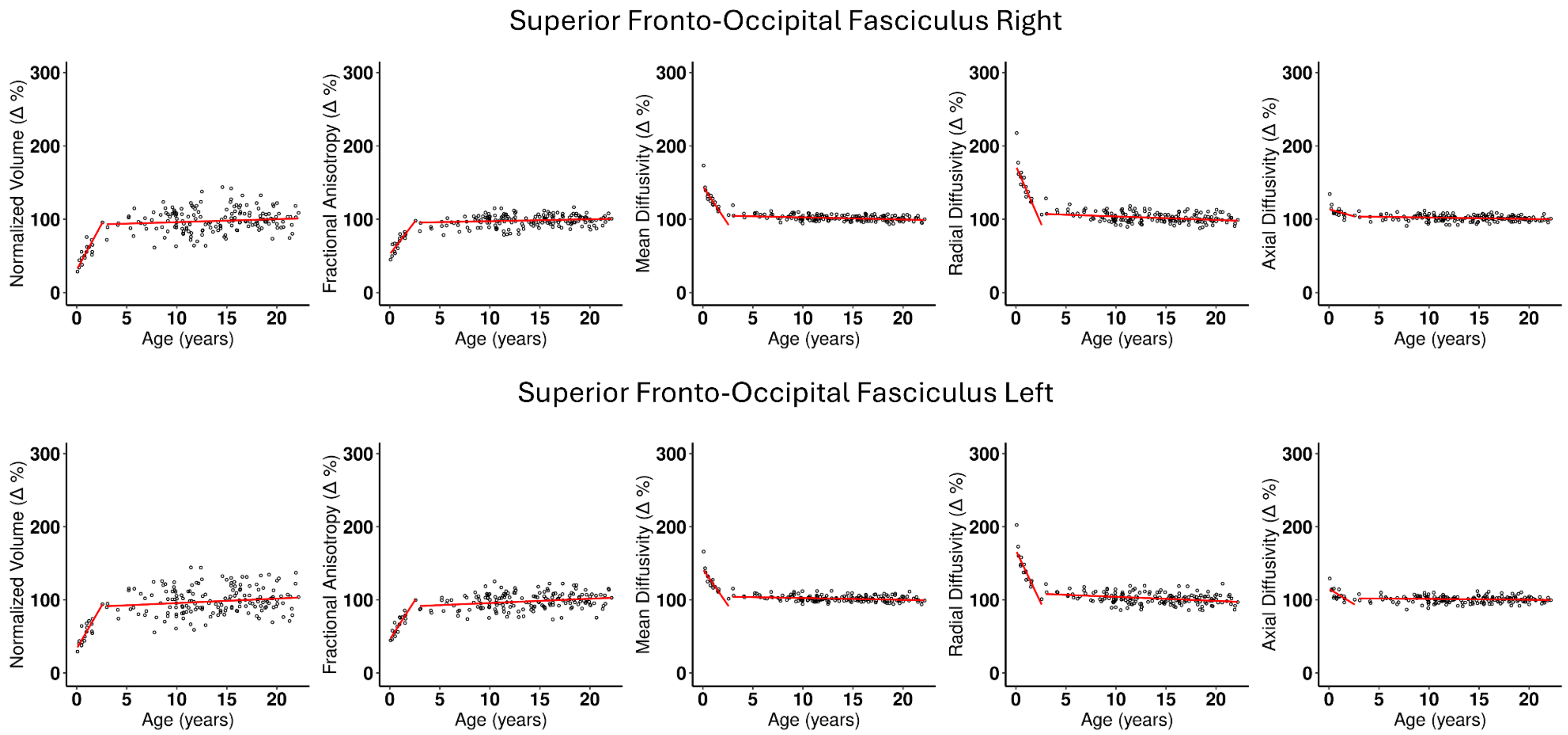
Superior Fronto-Occipital Fasciculus Right
Superior Fronto-Occipital Fasciculus Left
Normalized Volume (Δ %)
Fractional Anisotropy (Δ %)
Mean Diffusivity (Δ %)
Radial Diffusivity (Δ %)
Axial Diffusivity (Δ %)
Age (years)
0
100
200
300
5
10
15
20

(N)

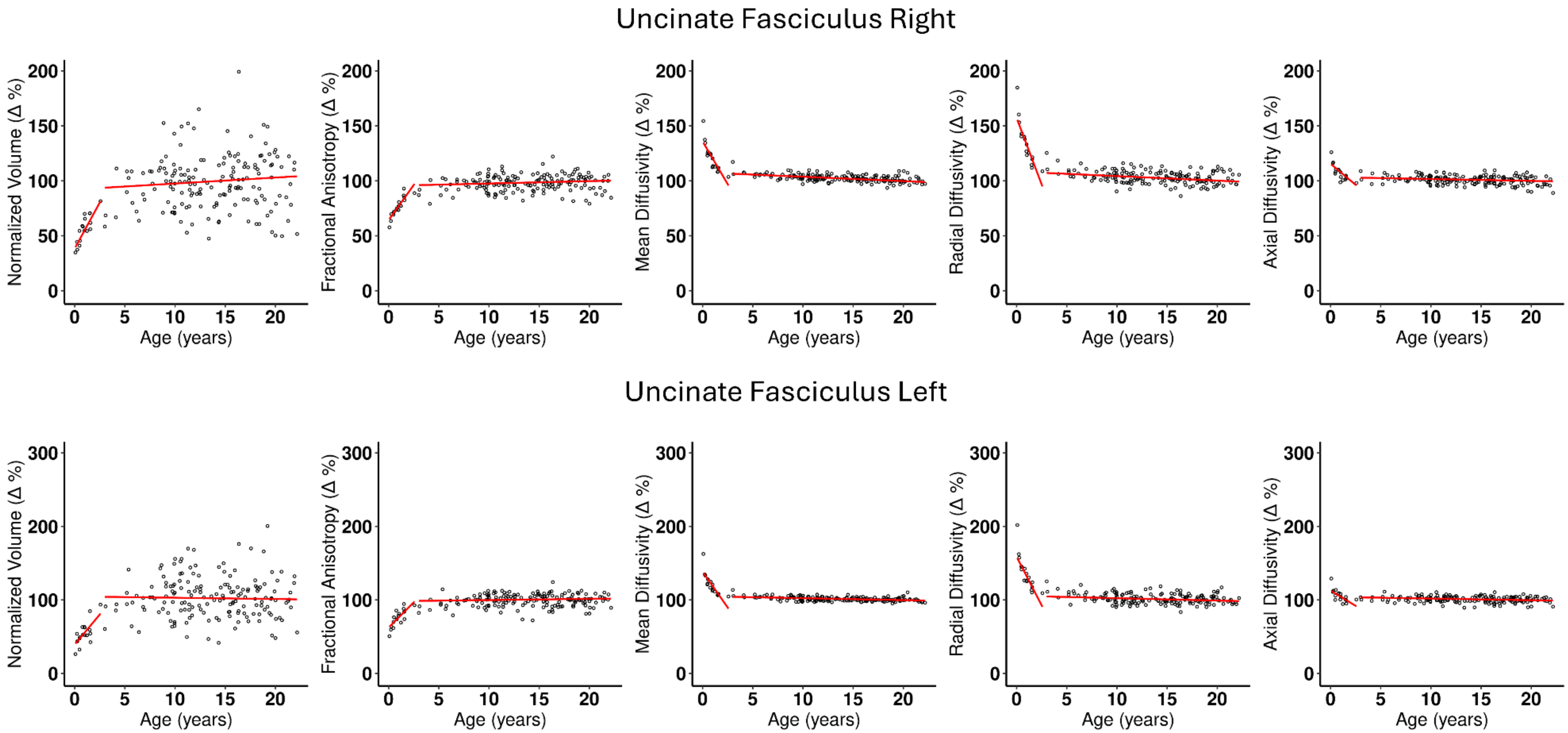

Uncinate Fasciculus Right
Normalized Volume (Δ %)
Fractional Anisotropy (Δ %)
Mean Diffusivity (Δ %)
Radial Diffusivity (Δ %)
Axial Diffusivity (Δ %)
Age (years)
Uncinate Fasciculus Left
Normalized Volume (Δ %)
Fractional Anisotropy (Δ %)
Mean Diffusivity (Δ %)
Radial Diffusivity (Δ %)
Axial Diffusivity (Δ %)
Age (years)

(O)

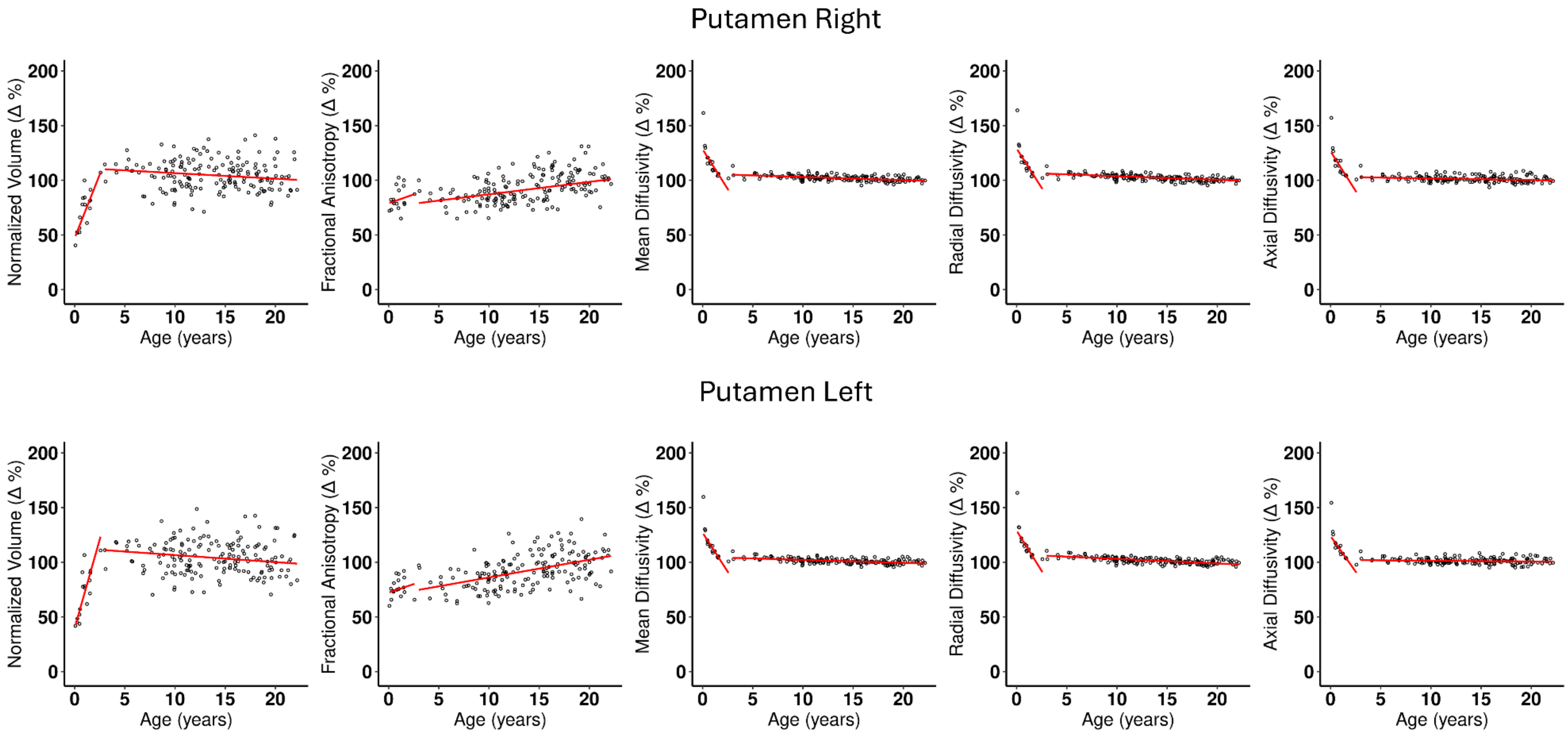

Putamen Right
Normalized Volume (Δ %)
Fractional Anisotropy (Δ %)
Mean Diffusivity (Δ %)
Radial Diffusivity (Δ %)
Axial Diffusivity (Δ %)
200
150
100
50
0
0
5
10
15
20
Age (years)
Putamen Left
Normalized Volume (Δ %)
Fractional Anisotropy (Δ %)
Mean Diffusivity (Δ %)
Radial Diffusivity (Δ %)
Axial Diffusivity (Δ %)
Age (years)

(P)

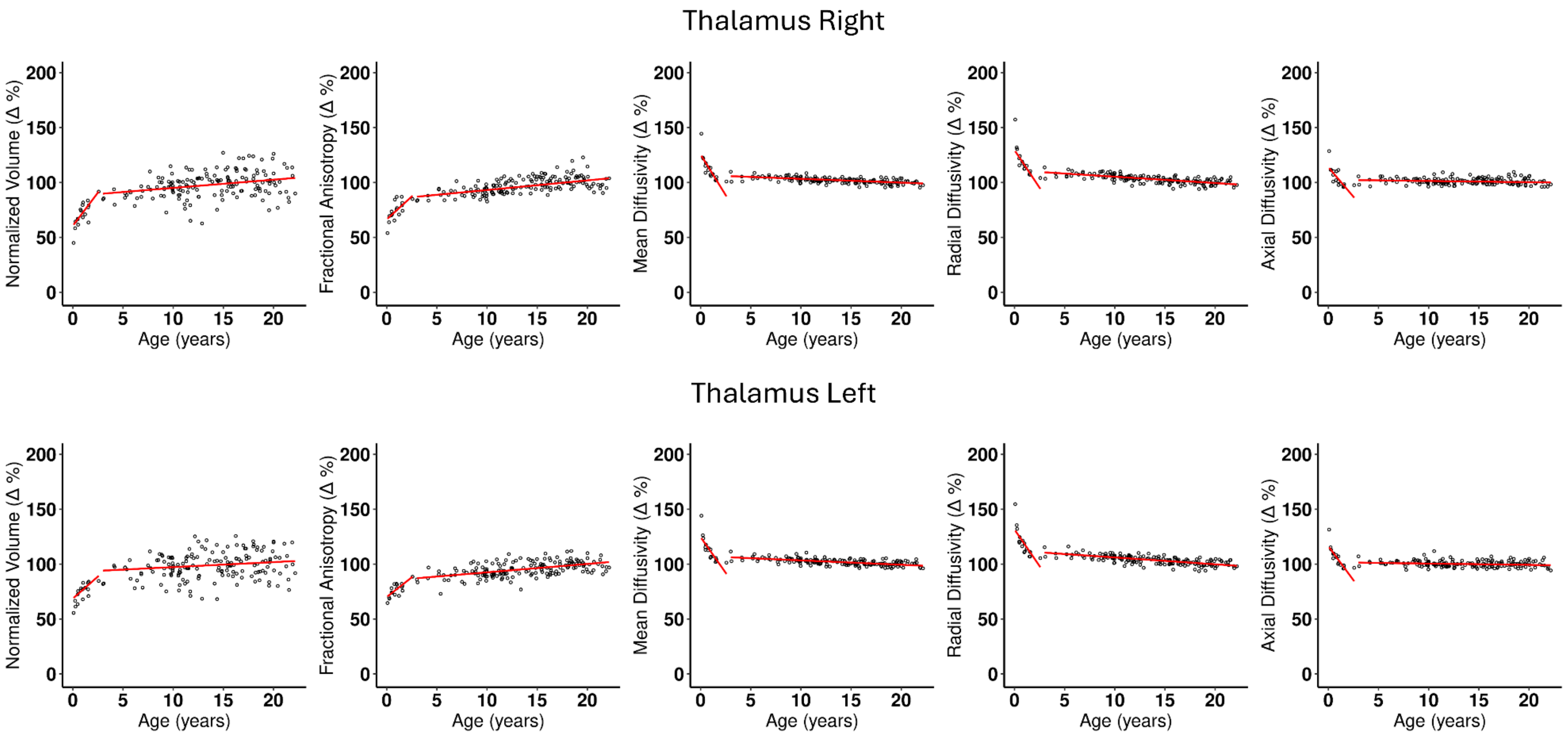

Figure S.5: (A) – (N) shows the scatterplots of each metric evaluated versus age for WM pathway ROIs not shown in the main paper. Each subject is represented by a dot in the plot. The x axis is the age distribution in years and the y axis is the normalized relative measurement with respect to the adult brain values, where the value for the adult brain is represented by 100%. The columns from left to right are Normalized (Δ%) for volume, Fractional anisotropy, Mean Diffusivity, Radial Diffusivity, and Axial Diffusivity. The two broken red lines in the plot are added after performing a nonparametric linear regression following the partition of the data into two groups around the 'change-point' age (as detailed in S.2 section of the supplement). Slope of the first segment is Slope1, and for segment 2 is Slope2. These scatter plots show graphically the information tabulated in table 1. The plots are organized by pathway groups as follows: **Commissural:** (A) Pontine Crossing Tract ; **Cerebellar:** (B) Inferior Cerebellar Peduncle and (C) Superior Cerebellar Peduncle; **Projection:** (D) Cerebral Peduncle, (E) Medial Lemniscus, (F) Posterior Limb of Internal Capsule, and (G)Retro-lenticular part of Internal Capsule; **Association:** (H) External Capsule, (I) Cingulum Cingulate Gyrus, (J) Cingulum Hippocampus, (K) Fornix Cres Stria Terminalis, Fornix,

(L) Superior Longitudinal Fasciculus, (M) Superior Fronto-Occipital Fasciculus, and (N) Uncinate Fasciculus. In addition, (O) and (P) show plots for Putamen and Thalamus.

REFERENCES

1. Fasola S, Muggeo VMR, Küchenhoff H. A heuristic, iterative algorithm for change-point detection in abrupt change models. Computational Statistics. 2018;33(2):997-1015.
2. Muggeo VMR. Estimating regression models with unknown break-points. Statistics in Medicine. 2003;22(19):3055-71.
3. Muggeo V. Segmented: An R Package to Fit Regression Models With Broken-Line Relationships. R News. 2008;8:20-5.
4. R Core Team. R: A language and environment for statistical computing. R Foundation for Statistical Computing, Vienna, Austria. URL: https://www.R-project.org/. 2024.
5. Christopoulos D. On the Efficient Identification of an Inflection Point. INTERNATIONAL JOURNAL OF MATHEMATICS AND SCIENTIFIC COMPUTING (ISSN: 2231-5330). 2016;6.
6. Christopoulos D. Introducing Unit Invariant Knee (UIK) As an Objective Choice for Elbow Point in Multivariate Data Analysis Techniques. SSRN Electronic Journal. 2016.
7. Christopoulos DT. Developing methods for identifying the inflection point of a convex/concave curve. arXiv preprint arXiv:12065478. 2012.
8. Koenker R. Quantile Regression. Cambridge: Cambridge University Press; 2005.
9. Koenker R, Bassett G. Regression Quantiles. Econometrica. 1978;46(1):33-50.
